# Galectin-9-based immune risk score model helps to predict relapse in stage I–III small cell lung cancer

**DOI:** 10.1136/jitc-2020-001391

**Published:** 2020-10-20

**Authors:** Peixin Chen, Liping Zhang, Wei Zhang, Chenglong Sun, Chunyan Wu, Yayi He, Caicun Zhou

**Affiliations:** 1 Department of Medical Oncology, Shanghai Pulmonary Hospital, Tongji University Medical School Cancer Institute, Tongji University School of Medicine, No 507 Zhengmin Road, Shanghai 200433, China; 2 Tongji University, No 1239 Siping Road, Shanghai 200433, China; 3 Department of Pathology, Shanghai Pulmonary Hospital, Tongji University Medical School Cancer Institute, Tongji University School of Medicine, No 507 Zhengmin Road, Shanghai 200433, China

**Keywords:** lung neoplasms, tumor microenvironment, lymphocytes, tumor-infiltrating, programmed cell death 1 receptor, T-lymphocytes

## Abstract

**Background:**

For small cell lung cancer (SCLC) therapy, immunotherapy might have unique advantages to some extent. Galectin-9 (Gal-9) plays an important role in antitumor immunity, while little is known of its function in SCLC.

**Materials and methods:**

By mean of immunohistochemistry (IHC), we tested the expression level of Gal-9 and other immune markers on both tumor cells and tumor-infiltrating lymphocytes (TILs) in 102 surgical-resected early stage SCLC clinical samples. On the basis of statistical analysis and machine learning results, the Gal-9-based immune risk score model was constructed and its predictive performance was evaluated. Then, we thoroughly explored the effects of Gal-9 and immune risk score on SCLC immune microenvironment and immune infiltration in different cohorts and platforms.

**Results:**

In the SCLC cohort for IHC, the expression level of Gal-9 on TILs was statistically correlated with the levels of program death-1 (p=0.001), program death-ligand 1 (PD-L1) (p<0.001), CD3 (p<0.001), CD4 (p<0.001), CD8 (p<0.001), and FOXP3 (p=0.047). High Gal-9 protein expression on TILs indicated better recurrence-free survival (30.4 months, 95% CI: 23.7–37.1 vs 39.4 months, 95% CI: 31.6–47.3, p=0.009). The immune risk score model which consisted of Gal-9 on TILs, CD4, and PD-L1 on TILs was established and validated so as to differentiate high-risk or low-risk patients with SCLC. The prognostic predictive performance of immune risk score model was better than single immune biomarker (area under the curve 0.671 vs 0.621–0.644). High Gal-9-related enrichment pathways in SCLC were enriched in immune system diseases and rheumatic disease. Furthermore, we found that patients with SCLC with low immune risk score presented higher fractions of activated memory CD4 T cells than patients with high immune risk score (p=0.048).

**Conclusions:**

Gal-9 is markedly related to tumor-immune microenvironment and immune infiltration in SCLC. This study emphasized the predictive value and promising clinical applications of Gal-9 in stage I–III SCLC.

## Introduction

Lung cancer is the most common malignancy around the world, and the primary cause of cancer-associated deaths.^1 2^ Small cell lung cancer (SCLC), accounting for around 10%–15% in all pathology types of primary lung cancers, is known for its high degree of malignancy, low differentiation, rapid progression and poor prognosis.^1–3^ Most of patients with SCLC are first diagnosed with extensive disease (ED) beyond surgical indications.^4^ In the past decades, platinum-based chemotherapy, with or without radiotherapy, was still recommended as the standard first-line therapy for SCLC.^5–8^ However, about 40% of patients with SCLC remain insensitive to chemotherapy.^9–11^ Recently, immunotherapy showed certain advantages in the treatment of SCLC.^9 12–15^ Showing improvements in patient prognosis, atezolizumab, one of programmed death ligands-1 (PD-L1) inhibitors, was approved by the Food and Drug Administration in the treatment of ED-SCLC.^12^ Two phase III studies, CASPIAN and IMPOWER 133, also suggested that the survival time was prolonged when immunotherapy was added in traditional chemotherapy, comparing with chemotherapy alone.^9 12^ Nevertheless, in another phase III study, CheckMate 331, immunotherapy did not benefit survival as second-line treatment for patients with SCLC after progress from chemotherapy.^16^ Therefore, it is necessary to precisely select patients with SCLC who might benefit from immunotherapy.

Galectin-9 (Gal-9) is one of soluble lectins with two binding sites of β-galactoside with three classical isoforms.^17–19^ Gal-9 plays a significant role in innate and adaptive immunity. It is reported that Gal-9 could damage the function of some CD4 positive T cells which were also known as helper T lymphocytes (Th), and innate immune cells.^20 21^ Gal-9 also participated in the differentiation of induced T regulatory cells (iTregs).^22^ However, several former researches also showed the positive immunological effect of Gal-9. Gal-9 promoted the activity of various kinds of immune cells, such as dendritic cells, macrophages and natural killer (NK) cells.^23 24^ Recently, Gal-9 was a promising therapeutic target in various types of cancers. In lung cancer-bearing mice, Gal-9 promoted survival by inducing the differentiation of macrophages.^25^ The apoptosis of tumor cells induced by Gal-9 was observed in liver cancer and esophageal carcinoma.^26–28^ In our study, we aimed to reveal the expression patterns of Gal-9 on tumor cells and tumor-infiltrating lymphocytes (TILs) by immunohistochemistry (IHC) tests, as well as its connection with other immune markers in SCLC. We also conducted survival analysis comparing patients with different Gal-9 levels. Furthermore, we investigated how Gal-9 regulates the SCLC-immune microenvironment and immunophenotype by comprehensive bioinformatic analysis.

## Patients and methods

### Patients

From 2014 to 2018, 102 SCLC surgery specimens were collected from the Shanghai Pulmonary Hospital, China. Two independent reviewers screened the pathological types and surgical histology reports (Chunyan Wu and Liping Zhang). The tumor-node-metastasis staging system version 8th was applied. Samples were obtained following written informed consent from all participants.

### IHC for Gal-9

After dewaxing by xylene and alcohol, all formalin-fixed, paraffin-embedded tissue slides were rinsed with distilled water. Then, the target retrieval solution kit (DM828 or DM829, Dako) was used for antigen repairing. In order to reduce the background staining, we used 3% hydrogen peroxide. Primary antibodies (Galectin-9, NBP2-45619, Novusbio), and secondary antibodies which were goat-anti-Mouse/Rabbit IgG that labeled with horseradish peroxidase were applied standardly.

### The Gal-9 IHC cut-off value

Two independent pathologists (Chunyan Wu and Liping Zhang) reviewed all clinical samples (online supplemental table S1). Once discrepant evaluations were obtained, they reviewed together to arrive at consensus results. More than 30% staining was the cut-off of Gal-9 on TILs. On tumor cells, all positive stains of Gal-9 were regarded as positive. The screening process to find the best cut-off point was completed by survival analysis.^19 29^

10.1136/jitc-2020-001391.supp1 Supplementary data

### eXtreme Gradient Boosting and risk score models

We adapted the eXtreme Gradient Boosting (XGBoost) algorithm to construct XGBoost predictive models by various immune biomarkers and clinical features.^30^ As a machine-learning technique, XGBoost algorithm could work with the data of first and second derivatives to discovery non-linear relationship. It also could employ regularization item to control the overfitting and overly complex of predictive model, and provide the contribution of each feature to the outcome. To be specific, the corresponding formula of regularization item is as followed:

$$\Omega (f_{k})=\gamma T+\frac{1}{2}\lambda {\left\|w\right\|}^{2}$$

where T represents the number of leaves, W is defined as the magnitude of leaf weights. Both γ and λ are two penalty parameters that could respectively control penalty for T and W. By means of cross-validation, the penalty parameter is chosen. In the process of pruning, the threshold value of γ helps restrict the internal nodes of tree. During the process of smoothing, coefficient λ was added, thus finally avoid overfitting.

In the study, the whole cohort was randomly divided into the calibration subset and training subset which accounted for 70%. The final XGBoost survival models were composed of the top three predictive features and limited to the maximum depth of 6. Further, the process of model construction was repeated for 1000 times so as to fully use the sample information. The predictive value of the XGBoost model was visualized by the log-rank test. Then, we combined results of XGBoost model which ranked the relative importance of each signature and Cox multivariate model which offered coefficients of selected features to construct risk score models for patients with SCLC. The prognostic risk score equation of immune biomarkers was: immune risk score=(–0.550*Gal-9 on TILs)−(0.295*CD4)–(0.407*PD-L1 on TILs). The performance of risk score model was assessed by the areas under time-dependent receiver-operating characteristic (ROC) curves (AUCs). The larger the AUCs, the higher the quality of prognostic prediction of XGBoost predictive model.

### Clinical value of Gal-9 and risk score model in advanced SCLC

In order to further evaluate the predictive value of Gal-9 and immune risk score in advanced patients, we used the cBioportal Database (https://www.cbioportal.org). The enrolled cBioportal dataset must meet the following inclusion criteria^1^: mRNA sequencing for tumor tissues from patients with clinical stage IV SCLC,^2^ complete mRNA expression data,^3^ prognostic information from patients with clinical stage IV SCLC.

### Validation of Gal-9 expression in SCLC

In order to verify the expression of Lgals9 mRNA which encodes Gal-9 protein in SCLC cell lines and tumor tissues, we used the Cancer Cell Line Encyclopedia (CCLE) Database (https://portals.broadinstitute.org/ccle)^31^ and the Gene Expression Omnibus (GEO) Database (https://www.ncbi.nlm.nih.gov/geo/). As one of cancer-related databases, CCLE currently summarizes the expression level of more than 80,000 genes in total 1457 cancer cell lines. GEO is a public genome database which provides various kinds of gene expression data of corresponding study. The mRNA expression data from GEO were identified according to the following inclusion criteria^1^: mRNA sequencing for tumor tissues from patients with clinical SCLC,^2^ mRNA sequencing for normal tissue,^3^ complete mRNA expression data. The following exclusion criteria were considered^1^: insufficient data were available to compare gene expression,^2^ mRNA sequencing for animals or cell lines. After downloading suitable expression profiling from GEO, limma R package was used for screening differently expressed genes (DEGs) between tumor and controlled group.

### Gene Set Enrichment Analysis

In order to explore different biological pathways between high Gal-9 expression group and low Gal-9 expression group, the Gene Set Enrichment Analysis (GSEA) software (V.4.0.3) was employed.^32^ We divided the mRNA expression dataset of GEO into two groups evenly based on the Gal-9 expression level and kept all parameters in GSEA set at their defaults. The network between Gal-9 and Gal-9-related genes with strong correlation (>0.6) was visualized by Cytoscape software (V.3.7.1; https://cytoscape.org/).^33^

### The landscape of immune infiltration in patients with SCLC

To investigate the landscape of immune infiltration in patients with SCLC with high and low immune risk, we applied CIBERSORT method to the mRNA expression profile. As one of online databases for immune-infiltration analysis, CIBERSORT provides relative proportion of 22 human immune cell types in tumor tissues on the basis of deconvolution method.^34^ LM22 is a leukocyte gene signature matrix with high sensitivity and specificity for estimating 22 human immune phenotypes, including naive B cells, memory B cells, CD8 T cells, different CD4 T cell types, Tregs, NK cells, plasma cells, monocytes, three macrophages types, and dendritic cells. According to the calculation results of immune risk score based on the expression level of Lgals9, CD4 and CD274 that encode PD-L1, 23 SCLC clinical samples were divided into high-risk and low-risk group. Then, by combining CIBERSORT with LM22, the assessment of the component of 22 immune cells in each clinical sample of high-risk and low-risk group was obtained.

### Statistical analysis

We evaluated the correlation analysis between Gal-9 status and clinical factors or program death-1 (PD-1)/PD-L1 by Χ^2^ tests. Through taking multiple characteristics into account, we used univariate and covariate logistic regression analysis for predicting Gal-9 expression. We also performed Cox regression analysis and Kaplan-Meier method, which helped compare the prognosis conditions of different groups. All statistical examines were two-sided, and the p value smaller than 0.05 was defined as statistical significance. By means of X-tile software (V.X86, Yale University, USA), we picked the best cut-off value of immune risk score. The statistical tool SPSS (V.22.0) and the R Programming Language (V.4.0.1) for Windows were installed for the data analysis.

## Results

### Patient features

There are 102 patients in total, with a mean age of 62.7. The majority of patients were under 70 years old (79/102, 77.5%). Among all enrolled participants, men (84/102, 82.4%) were more than women (18/102, 17.6%). There were 58 (56.9%) non-smokers, and 44 (43.1%) were smokers. All patients were stage I–III. In the cohort as a whole, stage I–II accounted for a little more than half (60, 58.8%) (table 1).

**Table 1:** Patients’ characteristics (n=102)

Characteristic	N (%)	Characteristic	N (%)
Gender		T stage*	
Female	18 (17.6)	1	40 (39.2)
Male	84 (82.4)	2	47 (46.1)
Age, median, years	62	3	13 (12.7)
<70	79 (77.5)	4	2 (2.0)
≥70	23 (22.5)	N stage*	
Smoking status		0	44 (43.2)
Non-smoker	58 (56.9)	1	23 (22.5)
Smoker	44 (43.1)	2	34 (33.3)
SCLC staging*		3	1 (1)
I–II	60 (58.8)	Metastasis†	
III	42 (41.2)	No	98 (96.1)
Postoperative treatment‡		Yes	4 (3.9)
Not receive	35 (34.3)		
Chemotherapy	40 (39.2)		
Radiotherapy	1 (0.01)		
Chemotherapy plus radiotherapy	26 (25.5)		

The cohort was also used to explore FOXP3 and HLA class II expression in SCLC.

*Pathological stage.

†Clinical stage: metastasis considered by clinical imaging before surgery.

‡All treatment after surgery.

N, lymph node; SCLC, small cell lung cancer; T, tumor.

More than half of patients received treatment after surgery, including chemotherapy alone (40/102, 39.2%), radiotherapy alone (1/102, 0.01%), and chemotherapy plus radiotherapy (26/102, 25.5%). All enrolled patients had pulmonary nodules which were highly suspected as malignant tumor by imaging examination, thus receiving surgery to further confirm pathological types and follow-up care. However, a total of 35 patients with SCLC were treated with surgery alone because of some practical reasons, such as the contraindication of chemotherapy, financial stress, and personal willingness of refusal of postoperative treatment. In addition, a small group of patients relapsed and died within a month, thus losing the chance of postoperative treatment.

### Gal-9 expression and its correlation with clinical and immune parameters

**Figure 1:**
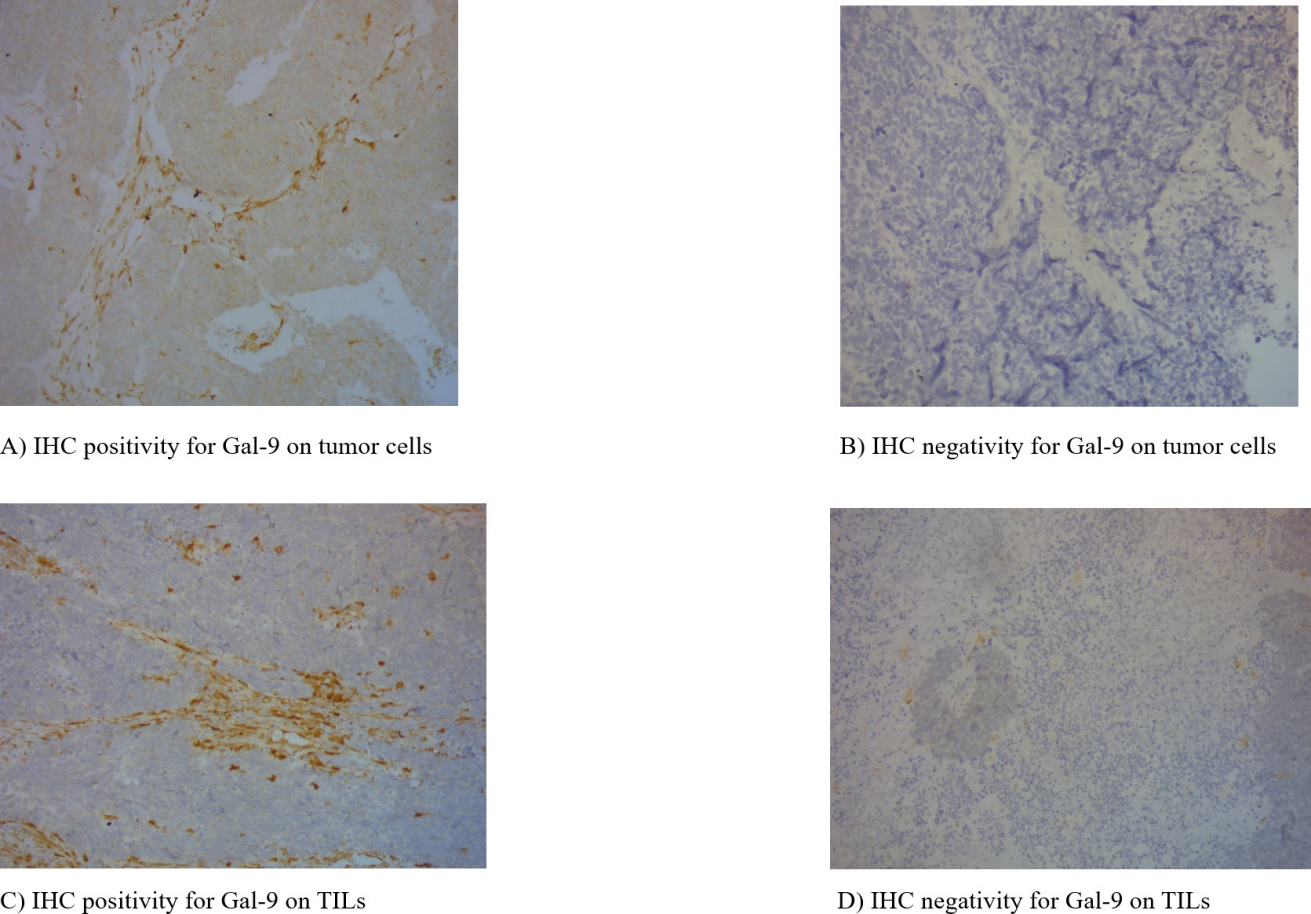
The expression of Gal-9 on cancer cells and TILs. Gal-9, galectin-9; IHC, immunohistochemistry; TILs, tumor-infiltrating lymphocytes.

In all specimens, 32 (31.4%) were positive Gal-9 expression on tumor cells and 28 (27.5%) were positive Gal-9 expression on TILs (figure 1). There was no significant correlation among clinical factors and Gal-9 level on tumor cells when gender, age, smoking status, metastasis status and SCLC staging were taken into consideration (p>0.05). Similarly, negative results were obtained in the Gal-9 level on TILs (p>0.05) (table 2).

**Table 2:** Relationship between galectin-9 (Gal-9) and clinical factors

Variables	Gal-9 expression on tumor cells			Gal-9 expression on TILs		
	Negative	Positive	P value	Negative	Positive	P value
Gender						
Female	12 (66.7%)	6 (33.3%)	0.843	15 (83.3%)	3 (16.7%)	0.259
Male	58 (69.0%)	26 (31.0%)		59 (70.2%)	25 (29.8%)	
Age (years)						
<70	57 (72.2%)	22 (27.8%)	0.155	56 (70.9%)	23 (29.1%)	0.486
≥70	13 (56.5%)	10 (43.5%)		18 (78.3%)	5 (21.7%)	
Smoking status						
Non-smoker	38 (65.5%)	20 (34.5%)	0.437	44 (75.9%)	14 (24.1%)	0.389
Smoker	32 (72.7%)	12 (27.3%)		30 (68.2%)	14 (31.8%)	
Metastasis						
Negative	68 (69.4%)	30 (30.6%)	0.588	71 (72.4%)	27 (27.6%)	1.000
Positive	2 (50.0%)	2 (50.0%)		3 (75.0%)	1 (25.0%)	
SCLC staging						
Stage I–II	41 (68.3%)	19 (31.7%)	0.939	41 (68.3%)	19 (31.7%)	0.254
Stage III	29 (69.0%)	13 (31.0%)		33 (78.6%)	9 (21.4%)	

SCLC, small cell lung cancer; TILs, tumor-infiltrating lymphocytes.

Through summarizing the correlation between Gal-9 on TILs and other immune biomarkers, we detected that the status of Gal-9 on TILs had widespread contacts with other immune checkpoints or immune cell level including PD-1 on TILs (p=0.001), PD-L1 on TILs (p<0.001), CD3 (p<0.001), CD4 (p<0.001), CD8 (p<0.001), and FOXP3 (p=0.047). However, results among different PD-L1 status on malignant cells, there was no significance of the TILs’ Gal-9 status in statistics (p=0.182). The p value, which was higher than 0.05, illustrated that the degree of Gal-9 expression on malignant cells was not significantly related to immune biomarkers that were taken into consideration (table 3).

**Table 3:** Relationship between galectin-9 (Gal-9) and other checkpoints

Variables	Gal-9 expression on tumor cells			Gal-9 expression on TILs		
	Negative	Positive	P value	Negative	Positive	P value
Gal-9 on tumor cells						
Negative	/	/	/	51 (72.9%)	19 (27.1%)	0.918
Positive				23 (71.9%)	9 (28.1%)	
PD-1 on TILs						
Negative	42 (65.6%)	22 (34.4%)	0.396	54 (84.4%)	10 (15.6%)	**0.001**
Positive	28 (73.7%)	10 (26.3%)		20 (52.6%)	18 (47.4%)	
PD-L1 on TILs						
Negative	47 (66.2%)	24 (33.8%)	0.423	61 (85.9%)	10 (14.1%)	**<0.001**
Positive	23 (74.2%)	8 (25.8%)		13 (41.9%)	18 (58.1%)	
PD-L1 on tumor cells						
Negative	67 (67.7%)	32 (32.3%)	0.550	73 (73.7%)	26 (26.3%)	0.182
Positive	3 (100.0%)	0 (0.0%)		1 (33.3%)	2 (66.7%)	
CD3						
Negative	37 (68.5%)	17 (31.5%)	0.980	52 (96.3%)	2 (3.7%)	**<0.001**
Positive	33 (68.8%)	15 (31.3%)		22 (45.8%)	26 (54.2%)	
CD4						
Negative	54 (70.1%)	23 (29.9%)	0.566	67 (87.0%)	10 (13.0%)	**<0.001**
Positive	16 (64.0%)	9 (36.0%)		7 (28.0%)	18 (72.0%)	
CD8						
Negative	51 (67.1%)	25 (32.9%)	0.571	65 (85.5%)	11 (14.5%)	**<0.001**
Positive	19 (73.1%)	7 (26.9%)		9 (34.6%)	17 (65.4%)	
FOXP3						
Negative	66 (68.8%)	30 (31.3%)	1.000	72 (75.0%)	24 (25.0%)	**0.047**
Positive	4 (66.7%)	2 (33.3%)		2 (33.3%)	4 (66.7%)	

Statistically significant data were marked with bold and underline.

PD-1, program death-1; PD-L1, program death-ligand 1; TILs, tumor-infiltrating lymphocytes.

### Logistic regression analysis of Gal-9 expression

By modifying relevant parameters, ORs and corresponding 95% CIs were summarized in online supplemental tables 2 and 3. On TILs, logistic regression analysis identified that the OR for Gal-9 status was 11.581 (95% CI, 2.093–64.083; p=0.005) when samples revealed CD3 positive compared with those revealed negative. Regretfully, none of other variables included had a statistically significant effect on SCLC cancer cells’ Gal-9 status.

### Relationship between Gal-9 status and prognosis in SCLC

**Figure 2:**
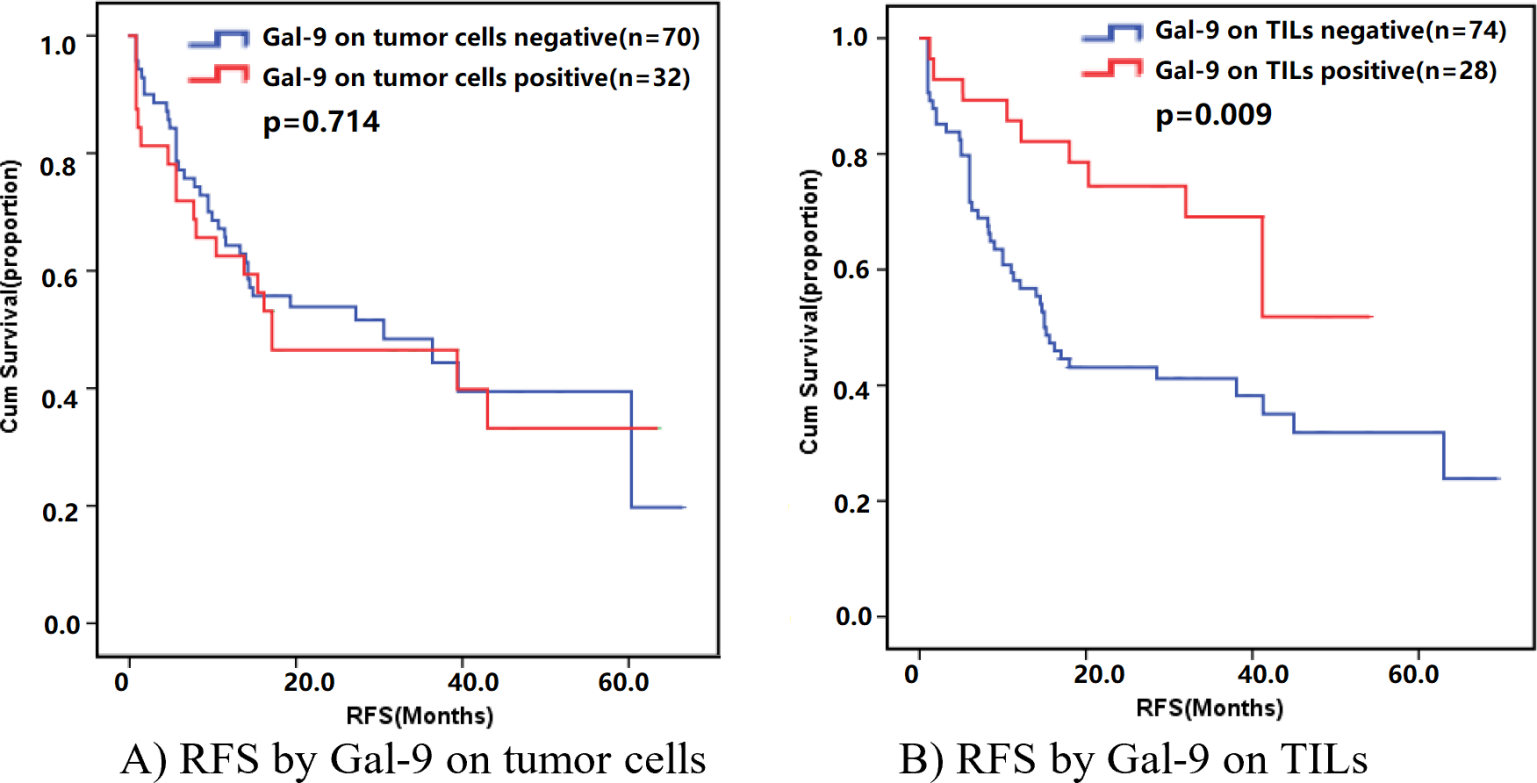
Survival analysis by Gal-9 level on tumor cells and TILs. Gal-9, galectin-9; RFS, recurrence-free survival; TILs, tumor-infiltrating lymphocytes.

In this study with 102 patients enrolled, the median recurrence-free survival (RFS) was 18.0 months, 56 (54.9%) patients had relapsed by the end of 2018. The median RFS calculated by the KM analysis was 32.0 months. In addition, the median RFS calculated by the KM analysis of stage I–II and stage III SCLC was 63.0 months, and 14.7 months, respectively. In all 60 patients in stage I–II, 25 (41.7%) reached the end event for RFS (median 19.0 months). The median RFS for all 42 patients with stage III SCLC was 15.0 months, among whom 31 (73.8%) had relapsed. With Kaplan-Meier method for time to relapse as the criterion standard, we analyzed the differences between positive Gal-9 and negative Gal-9 on TILs or tumor cells. We found that the positive Gal-9 on TILs demonstrated better RFS (RFS 30.4 months, 95% CI: 23.8–37.1 vs 39.4 months, 95% CI: 31.6–47.3, p=0.009). For the status of Gal-9 expression on tumor cells, the mean time of RFS was 32.0 months (95% CI, 22.2–41.8）in the positive group, and 35.1 months (95% CI, 28.0–42.3) for patients with SCLC with negative Gal-9 status. In spite of a difference between two datasets, no significance was showed in statistical terms (p=0.714; figure 2).

**Figure 3:**
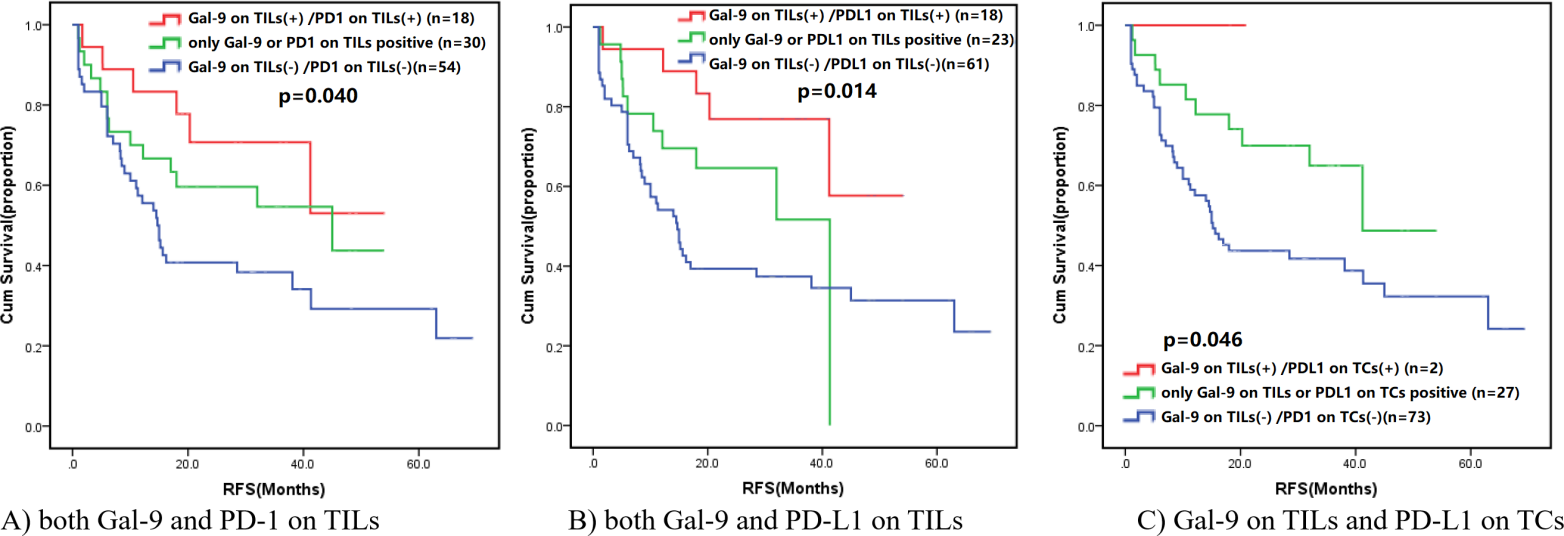
Survival analysis by Gal-9 level on TILs in combination with PD-1 or PD-L1. Gal-9, galectin-9; PD-1, program death-1; PD-L1, program death-ligand 1; RFS, recurrence-free survival; TCs, tumor cells; TILs, tumor-infiltrating lymphocytes.

We carried out the subgroup analysis based on Gal-9 level on TILs (figure 3 and online supplemental figure S1). It is worth noting that both Gal-9 and PD-1 on TILs positive (vs either Gal-9 and PD-1 on TILs positive or both Gal-9 and PD-1 on TILs negative; 39.4 months, 95% CI: 29.9–48.9 vs 33.1 months, 95% CI: 25.0–41.2 vs 28.8 months, 95% CI: 21.1–36.4, p=0.040), both Gal-9 and PD-L1 on TILs positive (vs either Gal-9 and PD-L1 on TILs positive or both Gal-9 and PD-L1 on TILs negative; 42.2 months, 95% CI: 33.5–50.9 vs 28.3 months, 95% CI: 21.3–35.3 vs 28.9 months, 95% CI: 21.7–36.1, p=0.014), both Gal-9 on TILs and CD3 positive (vs either Gal-9 on TILs and CD3 positive or both Gal-9 on TILs and CD3 negative; 40.4 months, 95% CI: 32.7–48.2 vs 35.4 months, 95% CI: 23.7–47.2 vs 27.9 months, 95% CI: 20.1–35.7, p=0.012), both Gal-9 on TILs and CD4 positive (vs either Gal-9 on TILs and CD4 positive or both Gal-9 on TILs and CD4 negative; 44.2 months, 95% CI: 35.9–52.5 vs 24.5 months, 95% CI: 16.7–32.3 vs 30.4 months, 95% CI: 23.5–37.4, p=0.017), either Gal-9 on TILs or CD8 positive (vs both Gal-9 on TILs and CD8 positive or both Gal-9 on TILs and CD8 negative; 45.5 months, 95% CI: 33.1–57.9 vs 41.3 months, 95% CI: 31.9–50.6 vs 27.9 months, 95% CI: 21.1–34.7, p=0.005) was notably correlated with longer RFS in SCLC. In spite of the significantly prognostic differences in the subgroups of Gal-9 on TILs in combination with PD-L1 on cancer cells (p=0.046) or FOXP3 (p=0.014), the double immune biomarkers positive group failed to fully reflect the objective fact for its limited sample size. The subgroup analysis of Gal-9 level on cancer cells in combination with PD-1, PD-L1, CD3, CD4, CD8, and FOXP3, respectively, showed no significant difference among different groups, which indicated the failure of Gal-9 on tumor cells in predicting the RFS in SCLC (online supplemental figure S2).

10.1136/jitc-2020-001391.supp2 Supplementary data

10.1136/jitc-2020-001391.supp3 Supplementary data

10.1136/jitc-2020-001391.supp4 Supplementary data

### Cox regression for survival analysis

Univariate and multivariate Cox regression models with categorical variables were established in sequence, for the purpose of adjusting for potential confounding characteristics and identifying prognostic factors. The HRs and their 95% CIs were calculated for assessment. By univariate Cox regression, SCLC staging (p=0.006) and Gal-9 level on TILs (p=0.012) were considered as the meaningfully predictive biomarkers for RFS. Multivariate Cox regression analysis further indicated that positive Gal-9 on TILs (vs negative GAL-9 on TILs; p=0.024, HR 0.436, 95% CI: 0.212–0.897), and SCLC stage I–II (vs SCLC stage III; p=0.014, HR 1.951, 95% CI: 1.146–3.322) were significantly related to better prognosis (table 4).

**Table 4:** Cox regression analysis

Variables	Univariate			Multivariate*			Multivariate†		
	HR	95% CI	P value	HR	95% CI	P value	HR	95% CI	P value
Gender (female vs male)	1.676	0.757–3.707	0.203						
Age (<70 vs ≥70)	1.770	0.997–3.143	0.051						
Smoking status (non-smoker vs smoker)	1.693	0.990–2.896	0.054						
Metastasis (negative vs positive)	0.877	0.214–3.605	0.856						
SCLC staging (I–II vs III)	2.111	1.243–3.586	**0.006**	1.951	1.146–3.322	**0.014**	1.836	1.077–3.131	**0.026**
Gal-9 on tumor cells (negative vs positive)	1.108	0.636–1.932	0.717						
Gal-9 on TILs (negative vs positive)	0.399	0.195–0.818	**0.012**	0.436	0.212–0.897	**0.024**			
Risk score (low vs high)	3.874	1.541–9.741	**0.004**				3.424	1.351–8.676	**0.009**

Statistically significant data were marked with bold and underline.

*Multivariate Cox regression analysis beyond risk score.

†Multivariate Cox regression analysis that included risk score.

Gal-9, galectin-9; SCLC, small cell lung cancer; TILs, tumor-infiltrating lymphocytes.

### Construction of the risk score model by XGBoost

**Figure 4:**
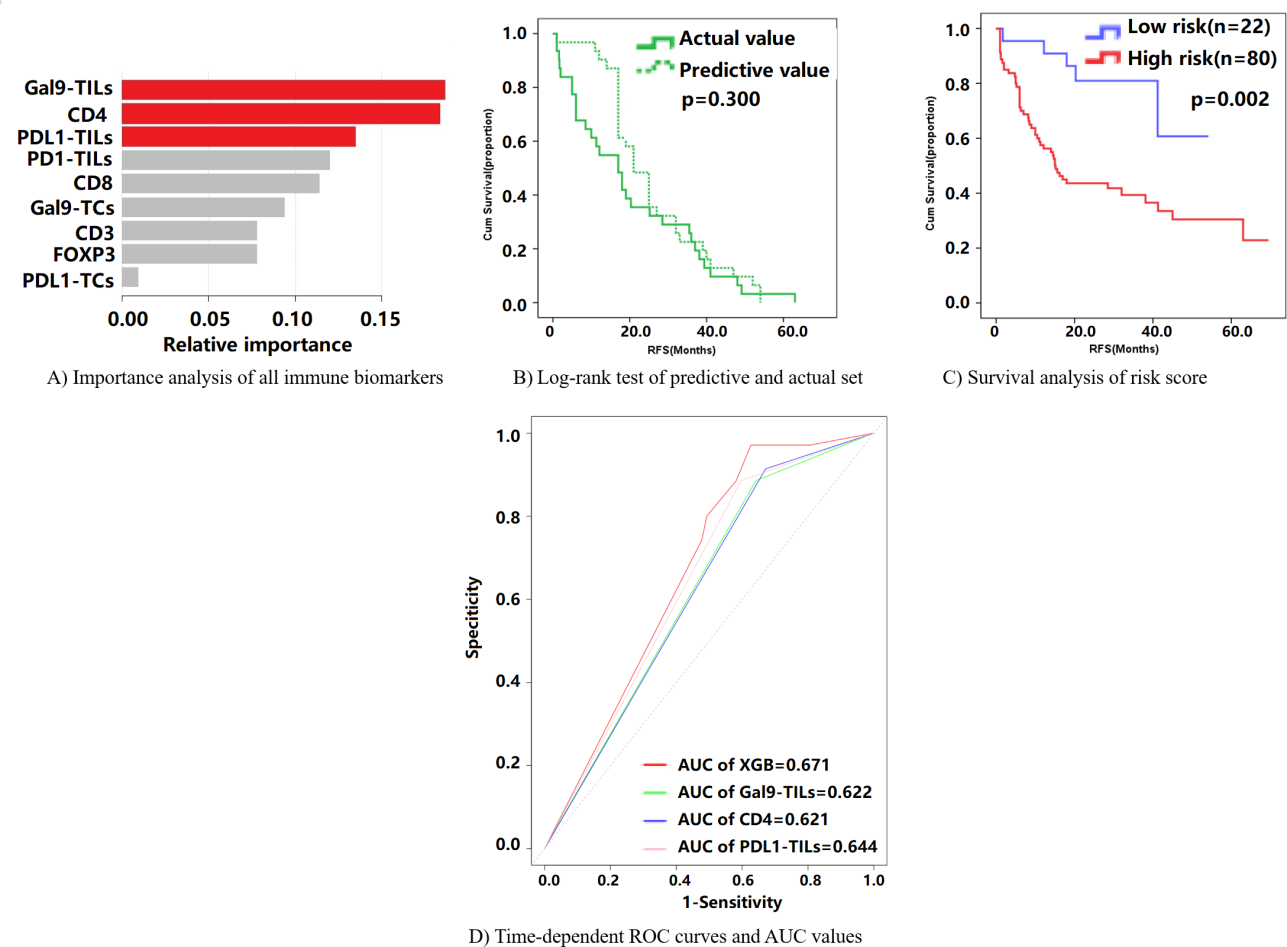
Prognostic performance of risk score model in SCLC. (A) Importance analysis of all immune biomarkers in SCLC by XGBoost algorithm. The diagram of feature importance to outcome illustrated that Gal-9 on TILs ranked first, CD4 second, and PD-L1 on TILs third. (B) Log-rank test results showed that the predictive curve was fitted well with the actual one in SCLC (21.000 months, 95% CI, 16.326–25.674, vs 17.000 months, 95% CI, 9.692–24.308, p=0.300). (C) Survival analysis of risk score. (D) Time-dependent ROC curves and AUC values for the risk score model and single immune biomarker. The AUC value for risk score model, Gal-9 on TILs, CD4, and PD-L1 on TILs were 0.671, 0.622, 0.621, 0.644, respectively. AUC, area under the curve; Gal-9, galectin-9; PD-1, program death-1; PD-L1, program death-ligand 1; RFS, recurrence-free survival; ROC, receiver operating characteristic; SCLC, small cell lung cancer; TILs, tumor-infiltrating lymphocytes; XGBoost, extreme gradient boosting algorithm.

Given the significant significance of subgroup analysis which all included Gal-9 level on TILs, we proposed the hypothesis that Gal-9 level on TILs had the meaningful interrelation with other immune biomarkers. To confirm this conjecture, XGBoost was first used for features selection. The diagram of feature importance to outcome which comprised all immune biomarkers illustrated that Gal-9 on TILs ranked first, CD4 second, and PD-L1 on TILs third (figure 4A). By incorporating top three variables, XGBoost results showed that the predictive curve was fitted well with the actual one (21.0 months, 95% CI: 16.3–25.7, vs 17.0 months, 95% CI: 9.7–24.3, p=0.300; figure 4B).

Therefore, Gal-9 on TILs, CD4, and PD-L1 on TILs were chosen to construct the prognostic risk score model for SCLC. The survival analysis demonstrated that the high-risk group contributed poorer prognosis (RFS, high risk 43.7 months, 95% CI: 35.9–51.5 vs low risk 29.9 months, 95% CI: 23.5–36.4, p=0.002; figure 4C). The significant difference between immune risk score and RFS was also suggested by univariate Cox regression (p=0.004, HR 3.874, 95% CI: 1.541–9.741; table 4). Considering that risk score covered the feature of Gal-9 status on TILs, the multivariate Cox regression model that included SCLC risk score and staging was established. Both risk score (p=0.009, HR 3.424, 95% CI: 1.351–8.676) and staging (p=0.026, HR 1.836, 95% CI: 1.077–3.131) were considered as independent prognostic features in SCLC (table 4). Furthermore, by means of time-dependent ROC analysis, immune risk score model obtained the best AUC value when compared with single immune biomarker. The AUC value for risk score model, Gal-9 on TILs, CD4, and PD-L1 on TILs were 0.671, 0.622, 0.621, 0.644, respectively (figure 4D), which highlighted that the risk score model performed better than other immune biomarkers in the prediction of prognosis in stage I–III SCLC.

### Clinical value of Gal-9 and risk score model in advanced SCLC

We downloaded the suitable SCLC dataset which contained 81 clinical SCLC samples from cBioportal Database.^35^ After data screening, the RNA sequencing (RNA-Seq) data of patients with stage IV SCLC were available for nine specimens (online supplemental table S4). For patients with SCLC in early and extensive stage, similar results of correlation analysis between Gal-9 and clinical factors or immune markers were obtained (online supplemental figure S3A and table S5). Gal-9 also especially showed significant correlation with PD-1 (p<0.001), PD-L1 (p<0.001), CD3 (p<0.001), CD4 (p<0.001), CD8 (p=0.04), and FOXP3 (p=0.002) in advanced SCLC. In addition, no significant correlation exhibited between Gal-9 and clinical features, including age (p=0.408) and sex (p=0.359).

10.1136/jitc-2020-001391.supp5 Supplementary data

Then, we also investigated the prognostic value of Gal-9 in nine patients with stage IV SCLC from public dataset. The survival analysis indicated that patients with extensive SCLC with higher Gal-9 expression level showed better overall survival (OS) than patients with lower Gal-9 expression (16.0 months, 95% CI: 7.4–24.6 vs 7.0 months, 95% CI: 2.1–11.9; p=0.122; online supplemental figure S3B). For better evaluating the performance of the risk score model, we applied it to patients with SCLC in extensive stage IV. The result supported that patients with advanced SCLC with higher risk score had shorter OS (vs lower risk score; 16.0 months, 95% CI: 7.4–24.6 vs 7.0 months, 95% CI: 2.1–11.9; p=0.122; online supplemental figure S4B).

10.1136/jitc-2020-001391.supp6 Supplementary data

### Validation of Gal-9 expression level in SCLC

We further verified the relative expression level of Gal-9 in both SCLC cell lines and tissues. The CCLE Database collected the mRNA expression level of Gal-9 coding gene, Lgals9, in 54 SCLC cell lines and 136 non-SCLC (NSCLC) cell lines. As the online supplemental figure S4A showed, Lgals9 was lowly expressed in the SCLC cell lines when compared with the NSCLC cell lines. In the GEO Database, both GSE43346 dataset which included expression profiles of 23 clinical SCLC tissues and 43 normal specimens, and GSE6044 dataset which was composed of 9 SCLC samples and 5 control subjects without cancer met the inclusion criteria, thus being included in this study. The significant differences of Lgals9 expression between SCLC and normal tissues were testified in the above two datasets (online supplemental figure S4B). Specifically, the expression level of Lgals9 in SCLC was lower than that of the controlled group (GSE43346, p=0.014; GSE6044, p=0.028).

### GSEA of Gal-9 expression-related pathways

**Figure 5:**
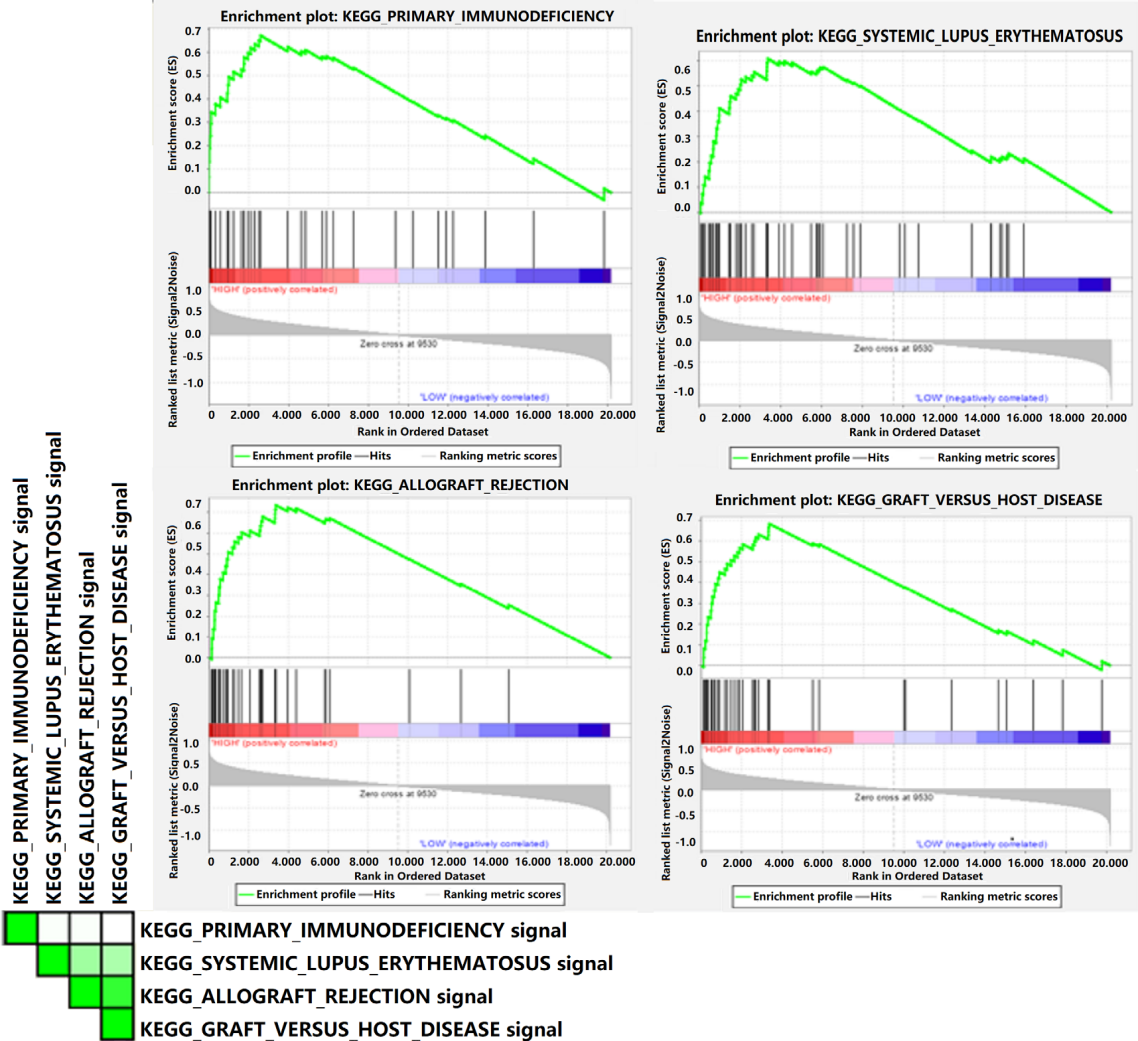
Gene set enrichment analysis of Gal-9 expression in SCLC. The top four significant enrichment plots in high Gal-9 expression group compared with that in low Gal-9 expression group. Gal-9, galectin-9; SCLC, small cell lung cancer.

For the sake of better understanding the biological pathways that significantly participated in group with high Gal-9 expression and investigating latent Gal-9-related genes, we performed GSEA and Cytoscape in GSE43346 dataset. Among a total of 174 gene sets which were upregulated or downregulated between two SCLC groups, 119 upregulated gene sets in the high Gal-9 expression group accounted for the largest proportion (119/174, 68.4%). Figure 5 demonstrated that the top four high Gal-9 expression-related pathways with enrichment scores >0.6 and false discovery rate <0.25 were as follows: “KEGG_PRIMARY_IMMUNODEFICIENCY”,“KEGG_SYSTEMIC_LUPUS_ERYTHEMATOSUS”, “KEGG_ALLOGRAFT_REJECTION”, and “KEGG_GRAFT_VERSUS_HOST_DISEASE”. A close relationship and high overlapping rate between the above four Gal-9 expression-related pathways were found (online supplemental figure S5A). A total of 15 genes overlapped in three of these pathways, indicating that they might played crucial roles in the high Gal-9 level. Among more than 20,000 genes, CD4 was also involved in the pathway which significantly enriched between different Gal-9 expressions. The results of leading edge analysis revealed that Jaccard values of numbers of the occurrences mainly concentrated on the range of 0–0.40 (online supplemental figure S5B). Further, we verified that 18 genes participated in mentioned high Gal-9-related pathways were differentially expressed between normal and SCLC tissues online supplemental figure S6A. In addition, the expression level of Gal-9 was moderately to highly related to that it of DEGs in the tumor microenvironment (online supplemental figure S6B). The correlation matrix also displayed that the expression level of all 18 DEGs had moderate correlations with each other. The network by Cytoscape visualized the strong correlation (>0.6) between all 18 DEGs and Lgals9 in SCLC (online supplemental figure S6C).

10.1136/jitc-2020-001391.supp7 Supplementary data

10.1136/jitc-2020-001391.supp8 Supplementary data

### Immune infiltration landscape between high-risk and low-risk group

**Figure 6:**
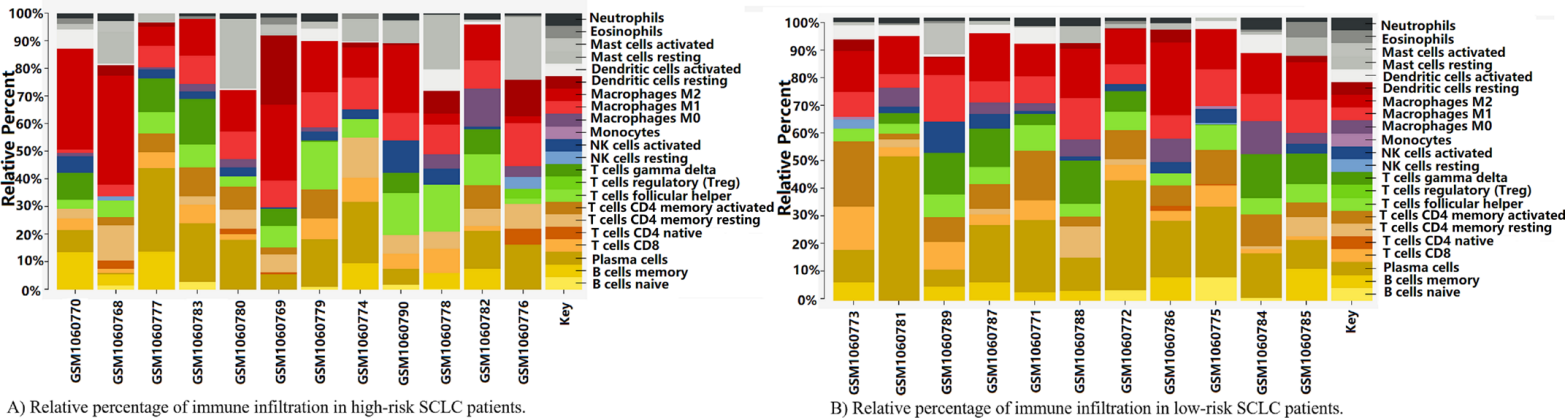
The landscapes of immune infiltration in patients with SCLC with high and low risk. (A) Relative percentage of immune infiltration in patients with high-risk SCLC. (B) Relative percentage of immune infiltration in patients with SCLC with low risk. NK, natural killer; SCLC, small cell lung cancer.

In the GEO SCLC cohort, we further analyzed the difference of immune infiltration condition between high-risk and low-risk score group by CIBERSORT and LM22. Two heatmaps separately depicted the detailed immune characteristics of 22 immune cells in patients with SCLC with high and low immune risk score (figure 6A, B). The relative percentage of TILs varied from sample to sample and summed up to 100%. Online supplemental figure S7A, B summarized the relationship between all immune cell proportions in two SCLC groups, while the correlation with each other was fairly modest. Then, we explored the significant differences of activated memory CD4 T cells between high-risk and low-risk group when the expression level of three prognostic biomarkers were all taken into account (p=0.048, online supplemental figure S7C). The low-risk group displayed considerable higher enrichment of activated memory CD4 T cells in comparison with high-risk group, indicating that the heterogeneity of immune cells in SCLC might act as a meaningful feature for outcome prediction. These results further verified the importance of the immune risk score model in terms of the tumor microenvironment and immune infiltration in SCLC.

10.1136/jitc-2020-001391.supp9 Supplementary data

## Discussion

The first aim of this study is to evaluate the status of Gal-9 expression on SCLC cancer cells and TILs. All clinical factors, which were included in the study, had no statistically significant influences on the level of Gal-9 on both TILs and tumor cells. However, our results revealed that Gal-9 expression level on TILs was related to the level of PD-1, PD-L1, immunocytes, and the recurrence time of patients with SCLC. More importantly, in comparison with positive Gal-9 expression on TILs, negative Gal-9 expression predicted early recurrence of patients with stage I−III SCLC. Then, we constructed the Gal-9-based risk score model which showed better prognostic performance in SCLC when compared with single biomarkers. We also tested the clinical values of Gal-9 and immune risk score model in patients with stage IV SCLC, which was consistent with our findings in patients with SCLC in early stage. In SCLC cell lines and tissues, we also verified the different expression levels of Lgals9 by public database. In addition, the meaningful results of GSEA and the Gal-9-based network helped to better understand the vital role of Gal-9 in SCLC and explore Gal-9-associated genes. The landscapes of immune infiltration in patients with SCLC with high and low risk suggested the immune heterogeneity in SCLC and further underlined the effects of the immune risk score model in tumor microenvironment.

The T-cell immunoglobulin mucin-3 (TIM-3) ligand Gal-9 was a member of mammalian lectins.^36–38^ Multiple types of cells, including thymocytes, leukocytes, endothelial cells and interferon-gamma-stimulated fibroblasts found Gal-9 expression, which revealed the significant role of Gal-9 in regulating immune processes.^36 37 39–42^ In vitro and vivo, Gal-9 induced death or suppressed function of T lymphocyte, including Th1 cells.^36 37 43^ Sehrawat *et al*^44^ found Gal-9 inhibited the immune response of effector T lymphocytes which expressed TIM-3 and CD8, and promoted the activity of FOXP3(+) Tregs. According to previous studies, Gal-9 could interact with 4-1BB (CD137) and DR3, suppress immunity and expand immunosuppressive Tregs, including CD8(+)/FOXP3(−) and CD4(+)/FOXP3(+) Tregs.^45 46^ These studies together indicated Gal-9 expression by immunocytes could affect innate and acquired immunity. Many published studies verified the distribution of Gal-9 protein among various malignant tissues, such as NSCLC, hematological malignancy, prostate cancer, as well as skin cancer.^19 38 47 48^ In spite of a considerable amount of studies focusing on the Gal-9 protein expression in cancer, few comprehensive data were available in SCLC. Considering its important function in antitumor immunity, it makes sense to describe the common status of Gal-9 on SCLC TILs and cancer cells.

Our test found protein expression of Gal-9 in SCLC. Our finding was in accord with the results of bioinformatics analysis, further enhancing high credibility of these results. We discovered Gal-9 on TILs was co-expressed with PD-1/PD-L1. Meanwhile, the Gal-9 expression on TILs was statistically related to the CD3, CD4, CD8, and FOXP3 expression. Gal-9 served as an influential factor during tumor development and metastasis. By using rat models of acute myelogenous leukemia, researchers found that Gal-9 knock-out decreased the accumulation of Tregs and promoted PD-1 and TIM-3 level on CD8(+) lymphocytes.^49^ In breast cancer cell lines, cell adhesion was promoted by Gal-9, from which Gal-9 showed its function in anti-metastasis.^50 51^ Moreover, Gal-9 exhibited its function in activating apoptosis of tumor cells by complicated signaling pathways. For myeloma cells, the activation of JNK and p38 MAP kinase pathways contributed to Gal-9-dependent apoptosis.^52^ For chronic myelogenous leukemia (CML) cells, transcription factor 3 played an important role in the Gal-9-induced cell death.^53^ Kuroda *et al*^53^ further confirmed the apoptosis-inducing effect of exogenous Gal-9 in CML cells. Nagahara *et al*^54^ found that Gal-9 promoted antitumor immunity by increasing the amount of CD8/TIM-3-positive T lymphocytes and CD86/TIM-3-positive dendritic cells. A couple of studies have demonstrated the medical potential of Gal-9 in cancer. And the complex mechanisms of the co-expression of Gal-9 and immune biomarkers were worthy of full illustration.

However, there was no correlation between Gal-9 status on cancer cells and other variables, including clinical factors and immune biomarkers. The same negative results were obtained between the Gal-9 expression on TILs and clinical factors. By means of literature consulting, we found that similar negative results were also obtained in other types of cancers, such as NSCLC and renal cell carcinoma.^19 55^ Regretfully, few researches were carried out to explore the mechanisms behind the lack of correlation between Gal-9 on cancer cells and other biomarkers to date. Considering the major function of Gal-9 and other biomarkers in tumor-related immune cells, we put forward the hypothesis that functional difference of these markers on TILs and cancer cells may result in the differential expression of Gal-9 among these cells, as well as the relative low relationship between Gal-9 on cancer cells and other biomarkers. In addition, the complex and dynamic tumor microenvironment may also have an influence on the relevance between Gal-9 and other specific factors. For further investigation and stronger credibility, fundamental researches as well as prospective and multicentered studies with larger sample size are necessary.

Furthermore, survival analysis was conducted in order to explore the relationship between Gal-9 and prognosis. Gal-9 expression level on tumor cells was of no value in predicting the relapse time in SCLC. However, patients with SCLC with positive Gal-9 on TILs contributed to longer progression-free survival than those had Gal-9 negative TILs. There are conflicting findings on the value of Gal-9 in predicting prognosis of a series of tumors. A meta-analysis demonstrated that Gal-9 overexpression was related to improved RFS in stomach cancer and patients with NSCLC.^56^ In addition, patients with positive Gal-9 expression also showed more satisfying prognosis in breast cancer and bladder cancer.^51 57^ Instead, elevated expression of Gal-9 led to a poor prognosis in patients with kidney carcinoma.^55 58^ In NSCLC, the expression of Gal-9 on both cancer cells and TILs was closely related to the clinical outcome.^19^ Patients with NSCLC who especially overexpressed Gal-9 on TILs displayed shorter RFS compared with those whose TILs had lower Gal-9 expression. Many reasons may lead to these contradictory findings. First, the study designs, technology, clinical endpoints, cut-off values, and sample sizes varied from study to study. The heterogeneity may be another main cause. There were heterogeneities in tumor types, locations, sizes, metastases, and stage. For example, in renal cell carcinoma，Jikuya *et al*^55^ indicated that Gal-9 was only related to poorer prognosis in patients in stage III–IV or grade 3. Moreover, different Gal-9 splice variants and receptors expression levels among various cancers may affect Gal-9 function and its prognostic value. It was reported that Gal-9 delta 5, instead of other Gal-9 variants, was the prognostic marker for NSCLC.^59^ The interaction between various immune biomarkers and immune cells might explain the inconsistent findings of outcome prediction ability of immune biomarkers in different researches. To fully use the power of Gal-9, it is worth to further investigate the specific mechanisms of Gal-9 in SCLC. We hold that the effect of antitumor and immunosuppressive should be balanced when applying Gal-9 in cancer treatment.

On SCLC TILs, the subgroup analysis indicated that positive Gal-9 protein in combination with PD-1 positive or PD-L1 positive was significantly related to better RFS. Similarly, positive outcome mentioned above was also found in the condition of Gal-9(+) in combination with CD3(+) or CD4(+) on TILs. In particular, for Gal-9 on TILs in combination with CD8, either Gal-9 on TILs or CD8 positive predicted improved RFS in SCLC. When Gal-9 was combined with CD8 in hepatocellular carcinoma,^60^ patients with high expression of both Gal-9 and CD8 tended to have longer survival, which was consistent with our finding in SCLC. In SCLC, higher expression of CD3 was supposed to be correlated with better survival, whereas PD-L1 overexpression had no or even opposing effect on survival.^61 62^ Conversely, Sun *et al*^63^ explored that patients with SCLC who expressed higher PD-L1 and CD8 protein had longer OS. The level of FOXP3 has shown its statistically prognostic value in SCLC, especially among patients without metastasis.^64^ However, in SCLC, few studies examined the prognostic value and clinical significance of Gal-9 in combination with other immune biomarkers or immune cells, including PD-1, PD-L1, CD3, CD4 and FOXP3. Our finding may fill the research gaps in this field and clarify the potential prognostic value of combining Gal-9 with PD-1, PD-L1, or several immune cells. Thus, the hypothesis of better prognostic value of Gal-9 in combination with other immune biomarkers was proposed.

The immune risk score model which was based on the results of machine learning XGBoost and Cox analysis intuitively demonstrated that integrating CD4 and PD-L1 on TILs could improve the prognostic prediction ability of Gal-9 on TILs in stage I–III SCLC. The log-rank test of predictive and actual dataset, the survival analysis of risk score in the whole cohort, and the time-dependent ROC curves illustrated higher accuracy and better performance of the Gal-9-based immune risk score model in comparison with single immune biomarkers. These observations highlighted that Gal-9 might regulate CD4 cells and PD-L1 on TILs. In addition, for the first time, we combined the expression level of Gal-9 with CD4 and PD-L1 on TILs to construct the immune risk score model, which provided a personalized scoring system for patients with stage I−III SCLC.

For patients with extensive stage IV SCLC, better OS was found in patients with high Gal-9 expression and low immune risk score, which was in compliance with our findings in patients with stage I–III SCLC. Nevertheless, no statistical difference was found between two groups, which may be due to several reasons. First, only nine patients with stage IV SCLC met the inclusive requirements and were enrolled in the survival analysis. The sample size was too small to assess the clinical value and application of Gal-9 in advanced SCLC systemically and objectively. Second, different results may be ascribed to the difference of clinical end point. Specifically, the construction of Gal-9-based immune risk score model was based on the RFS, while the end point of nine patients with extensive SCLC was OS. What is more, variability among study designs also affects the results. The Gal-9 expression of nine patients with extensive SCLC was measured by RNA-Seq, not by IHC. Given all these, the prognostic value of Gal-9 and immune risk score in advanced SCLC remains to be further elucidated in future researches.

Considering the effect of Lgals9 in tumor-immune microenvironment and immune infiltration, we performed GSEA, CIBERSORT and LM22 bioinformatic analysis. GSEA results showed that top four high Lgals9-related enrichment pathways in SCLC were “KEGG_PRIMARY_IMMUNODEFICIENCY”,“KEGG_SYSTEMIC_LUPUS_ERYTHEMATOSUS”, “KEGG_ALLOGRAFT_REJECTION”, and “KEGG_GRAFT_VERSUS_HOST_DISEASE”, with 18 Lgals9-related DEGs. The Lgals9-based network by Cytoscape showed extensive and complex correlation between Lgals9 and other molecules in tumor-immune microenvironment, including CD4, CD19, CD79A, CIS, IL2RG, FCGR2C, and FASLG. The CIBERSORT and LM22 results demonstrated the detailed landscapes of 22 immune cells infiltration in patients with SCLC with high and low immune risk score. The significant immune heterogeneity was found in activated memory CD4 T cells. Patients with SCLC with high immune risk score showed lower Gal-9 expression level, thus contributed to lower percentage of immune cells. These findings indicated that differential Gal-9 expression might result in variations in the SCLC-immune microenvironment and infiltration. A series of studies, in vitro and in vivo, affirmed the function of recombinant Gal-9 in promoting apoptosis, regulating tumor immunity, and inhibiting carcinoma progression.^52 53 65–68^ The pharmacokinetics of exogenous Gal-9 was investigated in mouse model,^69^ while less studies were available in humans. Thus, more researches and clinical trials were worthy of expected for exogenous Gal-9 which was considered as a potential therapeutic drug for SCLC. In addition, in consideration of the better RFS of patients with SCLC with positive Gal-9 and positive PD-L1, patients with SCLC might also benefit from exogenous Gal-9 plus PD-L1 inhibitors regimen.

Our study has its limitations. First, it is a retrospective study. Moreover, we draw our results and hypothesis by a rather small and single-centered cohort. A prospective and multicentered study is necessary in the future.

## Conclusions

In conclusion, the protein expression of Gal-9 on SCLC cancer cells and TILs was detected by IHC and validated by datasets. The co-expressed network of Gal-9 and PD-1, PD-L1, or immunocytes was also found on SCLC tumor cells and TILs. Furthermore, we constructed the immune risk score model by incorporating Gal-9 on TILs, CD4, and PD-L1 on TILs. Risk score was an independent prognostic factor for SCLC. Patients with SCLC with low immune risk score had longer postoperative recurrence time. This study highlighted the predictive value and promising clinical applications of Gal-9 in SCLC. Further investigation on Gal-9 is necessary so as to enhance our understanding of the underlying metabolic mechanism.

## Data Availability

Data are available upon reasonable request. All data relevant to the study are included in the article or uploaded as supplemental information. Emails could be sent to the address below to obtain the shared data: 1206339230@qq.com.

## References

[1] Siegel RL, Miller KD, Jemal A. Cancer statistics, 2019. CA Cancer J Clin 2019;69:7–34. 10.3322/caac.21551 30620402

[2] Fidler MM, Bray F. Global cancer inequalities. Front Oncol 2018;8:293. 10.3389/fonc.2018.00293 30155440 PMC6103267

[3] Byers LA, Rudin CM. Small cell lung cancer: where do we go from here? Cancer 2015;121:664–72. 10.1002/cncr.29098 25336398 PMC5497465

[4] Micke P, Faldum A, Metz T, et al. Staging small cell lung cancer: Veterans administration lung Study Group versus international association for the study of lung Cancer—what limits limited disease? Lung Cancer 2002;37:271–6. 10.1016/S0169-5002(02)00072-7 12234695

[5] Morabito A, Carillio G, Daniele G, et al. Treatment of small cell lung cancer. Crit Rev Oncol Hematol 2014;91:257–70. 10.1016/j.critrevonc.2014.03.003 24767978

[6] Früh M, De Ruysscher D, Popat S, et al. Small-Cell lung cancer (SCLC): ESMO clinical practice guidelines for diagnosis, treatment and follow-up. Ann Oncol 2013;24 Suppl 6:vi99–105. 10.1093/annonc/mdt178 23813929

[7] Stinchcombe TE, Gore EM. Limited-Stage small cell lung cancer: current chemoradiotherapy treatment paradigms. Oncologist 2010;15:187–95. 10.1634/theoncologist.2009-0298 20145192 PMC3227940

[8] Hanna N, Bunn PA, Langer C, et al. Randomized phase III trial comparing irinotecan/cisplatin with etoposide/cisplatin in patients with previously untreated extensive-stage disease small-cell lung cancer. J Clin Oncol 2006;24:2038–43. 10.1200/JCO.2005.04.8595 16648503

[9] Paz-Ares L, Dvorkin M, Chen Y, et al. Durvalumab plus platinum-etoposide versus platinum-etoposide in first-line treatment of extensive-stage small-cell lung cancer (Caspian): a randomised, controlled, open-label, phase 3 trial. Lancet 2019;394:1929–39. 10.1016/S0140-6736(19)32222-6 31590988

[10] Kuang P, Chen P, Wang L, et al. Rna sequencing analysis of small cell lung cancer reveals candidate chemotherapy insensitivity long noncoding RNAs and microRNAs. Ann Transl Med 2020;8:121. 10.21037/atm.2020.01.75 32175414 PMC7049041

[11] Chen S, He Y, Liu J, et al. Third-Generation TKI Resistance Due to SCLC Transformation: A Case Report and Brief Review]]&gt. OncoTargets and therapy 2019;12:11305–11. 10.2147/OTT.S228301 31908495 PMC6927588

[12] Horn L, Mansfield AS, Szczęsna A, et al. First-Line Atezolizumab plus chemotherapy in extensive-stage small-cell lung cancer. N Engl J Med 2018;379:2220–9. 10.1056/NEJMoa1809064 30280641

[13] Hellmann MD, Ott PA, Zugazagoitia J, et al. Nivolumab (nivo) ± ipilimumab (IPI) in advanced small-cell lung cancer (SCLC): first report of a randomized expansion cohort from CheckMate 032. JCO 2017;35:8503. 10.1200/JCO.2017.35.15_suppl.8503

[14] Ott PA, Elez E, Hiret S, et al. Pembrolizumab in patients with extensive-stage small-cell lung cancer: results from the phase Ib KEYNOTE-028 study. J Clin Oncol 2017;35:3823–9. 10.1200/JCO.2017.72.5069 28813164

[15] Gelsomino F, Leonetti A, Rihawi K, et al. Immune checkpoint inhibition in small cell lung cancer: a key to reach an unmet need? Transl Cancer Res 2017;6:S1484–8. 10.21037/tcr.2017.11.25

[16] Reck M, Vicente D, Ciuleanu T, et al. Efficacy and safety of nivolumab (nivo) monotherapy versus chemotherapy (chemo) in recurrent small cell lung cancer (SCLC): results from CheckMate 331. Annals of Oncology 2018;29:x43. 10.1093/annonc/mdy511.004

[17] Nagae M, Nishi N, Nakamura-Tsuruta S, et al. Structural analysis of the human galectin-9 N-terminal carbohydrate recognition domain reveals unexpected properties that differ from the mouse orthologue. J Mol Biol 2008;375:119–35. 10.1016/j.jmb.2007.09.060 18005988

[18] Cummings RD. T Cells Are Smad’ly in Love with Galectin-9. Immunity 2014;41:171–3. 10.1016/j.immuni.2014.08.001 25148018

[19] He Y, Jia K, Dziadziuszko R, et al. Galectin-9 in non-small cell lung cancer. Lung Cancer 2019;136:80–5. 10.1016/j.lungcan.2019.08.014 31454748

[20] Cao E, Zang X, Ramagopal UA, et al. T cell immunoglobulin mucin-3 crystal structure reveals a galectin-9-independent ligand-binding surface. Immunity 2007;26:311–21. 10.1016/j.immuni.2007.01.016 17363302

[21] Golden-Mason L, McMahan RH, Strong M, et al. Galectin-9 functionally impairs natural killer cells in humans and mice. J Virol 2013;87:4835–45. 10.1128/JVI.01085-12 23408620 PMC3624298

[22] Wu C, Thalhamer T, Franca RF, et al. Galectin-9-CD44 interaction enhances stability and function of adaptive regulatory T cells. Immunity 2014;41:270–82. 10.1016/j.immuni.2014.06.011 25065622 PMC4219323

[23] Dai S-Y, Nakagawa R, Itoh A, et al. Galectin-9 induces maturation of human monocyte-derived dendritic cells. J Immunol 2005;175:2974–81. 10.4049/jimmunol.175.5.2974 16116184

[24] Nobumoto A, Oomizu S, Arikawa T, et al. Galectin-9 expands unique macrophages exhibiting plasmacytoid dendritic cell-like phenotypes that activate NK cells in tumor-bearing mice. Clin Immunol 2009;130:322–30. 10.1016/j.clim.2008.09.014 18974023

[25] Kadowaki T, Arikawa T, Shinonaga R, et al. Galectin-9 signaling prolongs survival in murine lung-cancer by inducing macrophages to differentiate into plasmacytoid dendritic cell-like macrophages. Clin Immunol 2012;142:296–307. 10.1016/j.clim.2011.11.006 22177847

[26] Tadokoro T, Fujihara S, Chiyo T, et al. Induction of apoptosis by galectin-9 in liver metastatic cancer cells: in vitro study. Int J Oncol 2017;51:607–14. 10.3892/ijo.2017.4053 28656219

[27] Chiyo T, Fujita K, Iwama H, et al. Galectin-9 induces mitochondria-mediated apoptosis of esophageal cancer in vitro and in vivo in a xenograft mouse model. Int J Mol Sci 2019;20:2634. 10.3390/ijms20112634 PMC6600680 31146370

[28] Fujita K, Iwama H, OTO T, et al. Galectin-9 suppresses the growth of hepatocellular carcinoma via apoptosis in vitro and in vivo. Int J Oncol 2015;46:2419–30. 10.3892/ijo.2015.2941 25823465

[29] He Y, Yu H, Rozeboom L, et al. LAG-3 protein expression in non-small cell lung cancer and its relationship with PD-1/PD-L1 and tumor-infiltrating lymphocytes. J Thorac Oncol 2017;12:814–23. 10.1016/j.jtho.2017.01.019 28132868

[30] Chen T, Guestrin C. XGBoost: a scalable tree boosting system, 2016.

[31] Ghandi M, Huang FW, Jané-Valbuena J, et al. Next-Generation characterization of the cancer cell line encyclopedia. Nature 2019;569:503–8. 10.1038/s41586-019-1186-3 31068700 PMC6697103

[32] Subramanian A, Tamayo P, Mootha VK, et al. Gene set enrichment analysis: a knowledge-based approach for interpreting genome-wide expression profiles. Proc Natl Acad Sci U S A 2005;102:15545–50. 10.1073/pnas.0506580102 16199517 PMC1239896

[33] Shannon P et al. Cytoscape: a software environment for integrated models of biomolecular interaction networks. Genome Res 2003;13:2498–504. 10.1101/gr.1239303 14597658 PMC403769

[34] Newman AM, Liu CL, Green MR, et al. Robust enumeration of cell subsets from tissue expression profiles. Nat Methods 2015;12:453–7. 10.1038/nmeth.3337 25822800 PMC4739640

[35] George J, Lim JS, Jang SJ, et al. Comprehensive genomic profiles of small cell lung cancer. Nature 2015;524:47–53. 10.1038/nature14664 26168399 PMC4861069

[36] Wada J, Ota K, Kumar A, et al. Developmental regulation, expression, and apoptotic potential of galectin-9, a beta-galactoside binding lectin. J Clin Invest 1997;99:2452–61. 10.1172/JCI119429 9153289 PMC508086

[37] Wada J, Kanwar YS. Identification and characterization of galectin-9, a novel beta-galactoside-binding mammalian lectin. J Biol Chem 1997;272:6078–86. 10.1074/jbc.272.9.6078 9038233

[38] Türeci O, Schmitt H, Fadle N, et al. Molecular definition of a novel human galectin which is immunogenic in patients with Hodgkin's disease. J Biol Chem 1997;272:6416–22. 10.1074/jbc.272.10.6416 9045665

[39] Tsuboi Y, Abe H, Nakagawa R, et al. Galectin-9 protects mice from the Shwartzman reaction by attracting prostaglandin E2-producing polymorphonuclear leukocytes. Clin Immunol 2007;124:221–33. 10.1016/j.clim.2007.04.015 17560833

[40] Spitzenberger F, Graessler J, Schroeder HE. Molecular and functional characterization of galectin 9 mRNA isoforms in porcine and human cells and tissues. Biochimie 2001;83:851–62. 10.1016/s0300-9084(01)01335-9 11698107

[41] Thijssen VL, Hulsmans S, Griffioen AW. The galectin profile of the endothelium: altered expression and localization in activated and tumor endothelial cells. Am J Pathol 2008;172:545–53. 10.2353/ajpath.2008.070938 18202194 PMC2312370

[42] Asakura H, Kashio Y, Nakamura K, et al. Selective eosinophil adhesion to fibroblast via IFN-gamma-induced galectin-9. J Immunol 2002;169:5912–8. 10.4049/jimmunol.169.10.5912 12421975

[43] Zhu C, Anderson AC, Schubart A, et al. The Tim-3 ligand galectin-9 negatively regulates T helper type 1 immunity. Nat Immunol 2005;6:1245–52. 10.1038/ni1271 16286920

[44] Sehrawat S, Reddy PBJ, Rajasagi N, et al. Galectin-9/TIM-3 interaction regulates virus-specific primary and memory CD8+ T cell response. PLoS Pathog 2010;6:e1000882. 10.1371/journal.ppat.1000882 20463811 PMC2865527

[45] Madireddi S, Eun S-Y, Mehta AK, et al. Regulatory T cell-mediated suppression of inflammation induced by DR3 signaling is dependent on galectin-9. J Immunol 2017;199:2721–8. 10.4049/jimmunol.1700575 28877989 PMC5659314

[46] Madireddi S, Eun S-Y, Lee S-W, et al. Galectin-9 controls the therapeutic activity of 4-1BB–targeting antibodies. J Exp Med 2014;211:1433–48. 10.1084/jem.20132687 24958847 PMC4076583

[47] Laderach DJ, Gentilini LD, Giribaldi L, et al. A unique galectin signature in human prostate cancer progression suggests galectin-1 as a key target for treatment of advanced disease. Cancer Res 2013;73:86–96. 10.1158/0008-5472.CAN-12-1260 23108139

[48] Kageshita T, Kashio Y, Yamauchi A, et al. Possible role of galectin-9 in cell aggregation and apoptosis of human melanoma cell lines and its clinical significance. Int J Cancer 2002;99:809–16. 10.1002/ijc.10436 12115481

[49] Zhou Q, Munger ME, Veenstra RG, et al. Coexpression of Tim-3 and PD-1 identifies a CD8+ T-cell exhaustion phenotype in mice with disseminated acute myelogenous leukemia. Blood 2011;117:4501–10. 10.1182/blood-2010-10-310425 21385853 PMC3099570

[50] Irie A, Yamauchi A, Kontani K, et al. Galectin-9 as a prognostic factor with antimetastatic potential in breast cancer. Clin Cancer Res 2005;11:2962–8. 10.1158/1078-0432.CCR-04-0861 15837748

[51] Yamauchi A, Kontani K, Kihara M, et al. Galectin-9, a novel prognostic factor with antimetastatic potential in breast cancer. Breast J 2006;12:S196–200. 10.1111/j.1075-122X.2006.00334.x 16959001

[52] Kobayashi T, Kuroda J, Ashihara E, et al. Galectin-9 exhibits anti-myeloma activity through JNK and p38 MAP kinase pathways. Leukemia 2010;24:843–50. 10.1038/leu.2010.25 20200560

[53] Kuroda J, Yamamoto M, Nagoshi H, et al. Targeting activating transcription factor 3 by galectin-9 induces apoptosis and overcomes various types of treatment resistance in chronic myelogenous leukemia. Mol Cancer Res 2010;8:994–1001. 10.1158/1541-7786.MCR-10-0040 20571063

[54] Nagahara K, Arikawa T, Oomizu S, et al. Galectin-9 increases Tim-3+ dendritic cells and CD8^+^ T cells and enhances antitumor immunity via galectin-9-Tim-3 interactions. J Immunol 2008;181:7660–9. 10.4049/jimmunol.181.11.7660 19017954 PMC5886706

[55] Jikuya R, Kishida T, Sakaguchi M, et al. Galectin-9 expression as a poor prognostic factor in patients with renal cell carcinoma. Cancer Immunol Immunother 2020;69:2041–51. 10.1007/s00262-020-02608-6 32424467 PMC11027612

[56] Zhou X, Sun L, Jing D, et al. Galectin-9 expression predicts favorable clinical outcome in solid tumors: a systematic review and meta-analysis. Front Physiol 2018;9:452. 10.3389/fphys.2018.00452 29765332 PMC5939667

[57] Liu Y, Liu Z, Fu Q, et al. Galectin-9 as a prognostic and predictive biomarker in bladder urothelial carcinoma. Urol Oncol 2017;35:349–55. 10.1016/j.urolonc.2017.02.008 28347658

[58] Fu H, Liu Y, Xu L, et al. Galectin-9 predicts postoperative recurrence and survival of patients with clear-cell renal cell carcinoma. Tumor Biology 2015;36:5791–9. 10.1007/s13277-015-3248-y 25716202

[59] Schulkens IA, Heusschen R, van den Boogaart V, et al. Galectin expression profiling identifies galectin-1 and Galectin-9Δ5 as prognostic factors in stage I/II non-small cell lung cancer. PLoS One 2014;9:e107988. 10.1371/journal.pone.0107988 25259711 PMC4178059

[60] Sideras K, Biermann K, Verheij J, et al. PD-L1, Galectin-9 and CD8 ^+^ tumor-infiltrating lymphocytes are associated with survival in hepatocellular carcinoma. Oncoimmunology 2017;6:e1273309. 10.1080/2162402X.2016.1273309 28344887 PMC5353918

[61] Carvajal-Hausdorf D, Altan M, Velcheti V, et al. Expression and clinical significance of PD-L1, B7-H3, B7-H4 and TILs in human small cell lung cancer (SCLC). J Immunother Cancer 2019;7:65. 10.1186/s40425-019-0540-1 30850021 PMC6408760

[62] Zhao X, Kallakury B, Chahine JJ, et al. Surgical resection of SCLC: prognostic factors and the tumor microenvironment. J Thorac Oncol 2019;14:914–23. 10.1016/j.jtho.2019.01.019 30735815 PMC6510981

[63] Sun Y, Zhai C, Chen X, et al. Characterization of PD-L1 protein expression and CD8^+^ tumor-infiltrating lymphocyte density, and their associations with clinical outcome in small-cell lung cancer. Transl Lung Cancer Res 2019;8:748–59. 10.21037/tlcr.2019.10.09 32010554 PMC6976348

[64] Bonanno L, Pavan A, Dieci MV, et al. The role of immune microenvironment in small-cell lung cancer: distribution of PD-L1 expression and prognostic role of FOXP3-positive tumour infiltrating lymphocytes. Eur J Cancer 2018;101:191–200. 10.1016/j.ejca.2018.06.023 30077124

[65] Wiersma VR, de Bruyn M, van Ginkel RJ, et al. The glycan-binding protein galectin-9 has direct apoptotic activity toward melanoma cells. J Invest Dermatol 2012;132:2302–5. 10.1038/jid.2012.133 22572821 PMC3422695

[66] Kobayashi K, Morishita A, Iwama H, et al. Galectin-9 suppresses cholangiocarcinoma cell proliferation by inducing apoptosis but not cell cycle arrest. Oncol Rep 2015;34:1761–70. 10.3892/or.2015.4197 26260906

[67] Tadokoro T, Morishita A, Fujihara S, et al. Galectin-9: an anticancer molecule for gallbladder carcinoma. Int J Oncol 2016;48:1165–74. 10.3892/ijo.2016.3347 26797414

[68] Takano J, Morishita A, Fujihara S, et al. Galectin-9 suppresses the proliferation of gastric cancer cells in vitro. Oncol Rep 2016;35:851–60. 10.3892/or.2015.4452 26717877

[69] Seki M, Oomizu S, Sakata K-mei, et al. Galectin-9 suppresses the generation of Th17, promotes the induction of regulatory T cells, and regulates experimental autoimmune arthritis. Clinical Immunology 2008;127:78–88. 10.1016/j.clim.2008.01.006 18282810

