# Correction: Galectin-9-based immune risk score model helps to predict relapse in stage I–III small cell lung cancer

**DOI:** 10.1136/jitc-2020-001391corr1

**Published:** 2021-11-09

Chen P, Zhang L, Zhang W, *et al*. Galectin-9-based immune risk score model helps to predict relapse in stage I–III small cell lung cancer. *J Immunother Cancer* 2020;8:e001391. doi:10.1136/jitc-2020-001391.

This article has been corrected since it was first published. Additional footnotes have been added to Table 1 for clarification, and the following sentence has been added to the Results section: ‘The median RFS calculated by the KM analysis was 32.0 months. In addition, the median RFS calculated by the KM analysis of stage I–II and stage III SCLC was 63.0 months, and 14.7 months, respectively.’.

