# Supplementary material for: Galectin-9-based immune risk score model helps to predict relapse in stage I–III small cell lung cancer
Source: J Immunother Cancer. 2020 Oct 20;8(2):e001391. doi: 10.1136/jitc-2020-001391 (PMC7577067; doi:10.1136/jitc-2020-001391)
Supplement: Supplementary data [file jitc-2020-001391supp001.pdf]

Table S1. Characteristics of 102 SCLC patients.

						Pathology Review		Gal-9 expression on cancer cells			Gal-9 expression on TILs				
**Sample-ID**	**Age，years**	**Sex**	**Smoke**	**Postoperative treatment**	**Stage**	**Pathology review 1**	**Pathology review 2**	**Review 1**	**Review 2**	**Review 1**	**Review 1**	**Review 2**	**Review 1**	**RFS-Status**	**RFS, months**
1	55	male	non-smoker	chemotherapy	1	SCLC	SCLC	0	0	0	30	30	30	1	63
2	52	male	non-smoker	not receive	3	SCLC	SCLC	0	0	0	10	10	10	1	1.2
3	76	male	non-smoker	not receive	1	SCLC	SCLC	90	80	90	20	20	20	1	5
4	58	male	non-smoker	chemotherapy plus radiotherapy	2	SCLC	SCLC	0	0	0	30	30	30	0	69.4
5	55	male	non-smoker	chemotherapy	1	SCLC	SCLC	100	100	100	30	20	30	0	66.3
6	51	male	non-smoker	chemotherapy plus radiotherapy	3	SCLC	SCLC	70	90	80	5	5	5	1	6
7	76	male	smoker	chemotherapy plus radiotherapy	3	SCLC	SCLC	0	0	0	20	20	20	1	6
8	47	female	smoker	not receive	3	SCLC	SCLC	40	40	40	5	5	5	0	65.7
9	65	female	smoker	chemotherapy plus radiotherapy	3	SCLC	SCLC	0	0	0	30	30	30	0	62.2
10	72	male	smoker	chemotherapy plus radiotherapy	3	SCLC	SCLC	90	90	90	5	5	5	1	14.5
11	73	male	smoker	chemotherapy	3	SCLC	SCLC	90	90	90	5	5	5	1	45
12	56	male	smoker	chemotherapy plus radiotherapy	3	SCLC	SCLC	0	0	0	10	10	10	1	15.6
13	66	male	smoker	radiotherapy	3	SCLC	SCLC	0	0	0	50	50	50	1	32
14	58	male	smoker	not receive	3	SCLC	SCLC	90	90	90	1	1	1	1	1
15	68	female	non-smoker	chemotherapy	3	SCLC	SCLC	0	0	0	30	30	30	1	2
16	60	male	non-smoker	chemotherapy plus radiotherapy	2	SCLC	SCLC	0	0	0	5	5	5	0	54
17	67	male	non-smoker	chemotherapy	2	SCLC	SCLC	0	0	0	60	60	60	0	54.1
18	43	female	non-smoker	chemotherapy	2	SCLC	SCLC	0	0	0	50	50	50	0	49.1
19	76	male	non-smoker	not receive	3	SCLC	SCLC	100	100	100	100	100	100	1	1.2
20	53	male	non-smoker	not receive	1	SCLC	SCLC	50	50	50	5	5	5	1	1
21	60	male	smoker	chemotherapy plus radiotherapy	3	SCLC	SCLC	0	0	0	20	20	20	1	41.3
22	51	female	non-smoker	chemotherapy	3	SCLC	SCLC	90	90	90	5	5	5	1	8.2
23	52	male	non-smoker	chemotherapy	3	SCLC	SCLC	0	0	0	5	5	5	1	15.2
24	78	male	non-smoker	not receive	1	SCLC	SCLC	0	0	0	5	5	5	0	49
25	54	male	non-smoker	chemotherapy	3	SCLC	SCLC	0	0	0	40	40	40	1	5.2
26	74	male	non-smoker	chemotherapy plus radiotherapy	3	SCLC	SCLC	0	0	0	20	10	20	1	5
27	60	male	non-smoker	chemotherapy	2	SCLC	SCLC	0	0	0	50	50	50	0	48
28	68	female	non-smoker	chemotherapy	3	SCLC	SCLC	0	0	0	0	0	0	1	38.1
29	66	female	non-smoker	chemotherapy plus radiotherapy	2	SCLC	SCLC	80	90	90	10	10	10	0	48.3
30	73	male	non-smoker	chemotherapy	1	SCLC	SCLC	80	90	90	40	40	40	1	18
31	65	male	non-smoker	chemotherapy	2	SCLC	SCLC	50	60	50	10	10	10	0	48
32	67	male	smoker	not receive	1	SCLC	SCLC	0	0	0	60	60	60	1	1.7
33	73	female	non-smoker	chemotherapy	3	SCLC	SCLC	0	0	0	20	20	20	1	4.8
34	75	male	smoker	not receive	1	SCLC	SCLC	100	100	100	40	40	40	1	41.2
35	59	male	smoker	chemotherapy	2	SCLC	SCLC	90	90	90	10	10	10	0	45.3

36	75	male	smoker	chemotherapy	1	SCLC	SCLC	40	40	40	5	5	5	1	17
37	65	male	smoker	not receive	1	SCLC	SCLC	20	30	20	10	10	10	1	8.5
38	63	female	non-smoker	not receive	1	SCLC	SCLC	90	90	90	50	50	50	0	41
39	57	male	smoker	not receive	3	SCLC	SCLC	80	80	80	30	30	30	1	1
40	40	male	non-smoker	chemotherapy plus radiotherapy	1	SCLC	SCLC	0	0	0	30	30	30	1	6.3
41	42	male	smoker	not receive	2	SCLC	SCLC	90	90	90	20	20	20	1	1
42	58	male	non-smoker	chemotherapy	2	SCLC	SCLC	0	0	0	20	20	20	1	15
43	54	male	smoker	chemotherapy	3	SCLC	SCLC	0	0	0	40	40	40	0	41
44	72	male	smoker	chemotherapy plus radiotherapy	3	SCLC	SCLC	50	50	50	10	10	10	1	6
45	56	female	non-smoker	not receive	2	SCLC	SCLC	0	0	0	20	20	20	0	39.4
46	66	male	smoker	chemotherapy	1	SCLC	SCLC	0	0	0	30	30	30	1	8.3
47	70	male	smoker	chemotherapy	3	SCLC	SCLC	40	40	40	5	5	5	1	11
48	52	male	smoker	not receive	3	SCLC	SCLC	0	0	0	50	50	50	0	37
49	66	male	smoker	chemotherapy plus radiotherapy	3	SCLC	SCLC	0	0	0	5	5	5	1	2
50	77	male	smoker	chemotherapy plus radiotherapy	3	SCLC	SCLC	0	0	0	60	60	60	0	36
51	63	female	non-smoker	chemotherapy plus radiotherapy	3	SCLC	SCLC	0	0	0	5	5	5	1	14
52	74	male	smoker	chemotherapy plus radiotherapy	3	SCLC	SCLC	90	90	90	5	5	5	1	18
53	64	male	smoker	not receive	2	SCLC	SCLC	70	70	70	40	40	40	0	37.1
54	67	male	non-smoker	not receive	3	SCLC	SCLC	70	70	70	40	40	40	0	36
55	81	male	smoker	not receive	1	SCLC	SCLC	0	0	0	50	50	50	1	12.2
56	38	male	non-smoker	chemotherapy	1	SCLC	SCLC	90	90	90	60	60	60	0	35.5
57	63	male	smoker	not receive	2	SCLC	SCLC	0	0	0	50	50	50	0	35.2
58	76	male	non-smoker	chemotherapy	3	SCLC	SCLC	0	0	0	5	5	5	1	7
59	67	male	smoker	chemotherapy	3	SCLC	SCLC	0	0	0	80	80	80	1	20.3
60	74	male	smoker	chemotherapy plus radiotherapy	3	SCLC	SCLC	0	0	0	10	10	10	1	14.7
61	67	female	non-smoker	not receive	1	SCLC	SCLC	50	50	50	10	10	10	1	1.6
62	63	male	non-smoker	not receive	3	SCLC	SCLC	90	90	90	20	20	20	1	16.2
63	47	female	non-smoker	not receive	2	SCLC	SCLC	0	0	0	30	30	30	1	1
64	66	male	smoker	not receive	2	SCLC	SCLC	0	0	0	40	30	30	1	6
65	68	male	non-smoker	not receive	1	SCLC	SCLC	80	80	80	10	10	10	0	33.4
66	61	male	non-smoker	not receive	1	SCLC	SCLC	90	90	90	40	40	40	0	33
67	72	male	smoker	not receive	1	SCLC	SCLC	0	0	0	10	10	10	0	31
68	68	male	smoker	not receive	1	SCLC	SCLC	0	0	0	5	5	5	1	10
69	61	female	non-smoker	chemotherapy	1	SCLC	SCLC	0	0	0	80	80	80	0	29.2
70	49	male	smoker	chemotherapy plus radiotherapy	3	SCLC	SCLC	0	0	0	10	10	10	0	31.4
71	68	male	non-smoker	chemotherapy	3	SCLC	SCLC	0	0	0	5	5	5	1	28.5
72	54	male	non-smoker	chemotherapy plus radiotherapy	2	SCLC	SCLC	0	0	0	20	20	20	0	31
73	52	male	non-smoker	not receive	1	SCLC	SCLC	0	0	0	10	10	10	0	31.6
74	58	male	smoker	chemotherapy plus radiotherapy	3	SCLC	SCLC	0	0	0	5	5	5	0	31
75	63	male	smoker	chemotherapy	3	SCLC	SCLC	0	0	0	5	5	5	0	29.3

76	64	male	smoker	not receive	2	SCLC	SCLC	0	0	0	10	10	10	1	6
77	51	male	smoker	chemotherapy	2	SCLC	SCLC	0	0	0	10	10	10	1	15
78	61	male	smoker	chemotherapy	1	SCLC	SCLC	0	0	0	20	20	20	0	28
79	68	male	non-smoker	chemotherapy	3	SCLC	SCLC	0	0	0	40	40	40	0	28.2
80	62	male	smoker	not receive	2	SCLC	SCLC	0	0	0	5	5	5	1	1
81	63	female	non-smoker	chemotherapy	3	SCLC	SCLC	0	0	0	20	20	20	0	25.2
82	55	male	non-smoker	chemotherapy plus radiotherapy	3	SCLC	SCLC	0	0	0	10	10	10	1	10
83	66	male	smoker	chemotherapy	1	SCLC	SCLC	0	0	0	60	60	60	1	10.5
84	73	male	smoker	not receive	2	SCLC	SCLC	0	0	0	10	10	10	1	1
85	63	male	non-smoker	chemotherapy	1	SCLC	SCLC	0	0	0	50	50	50	0	21.6
86	65	male	non-smoker	chemotherapy	1	SCLC	SCLC	0	0	0	80	80	80	0	21
87	69	male	smoker	not receive	1	SCLC	SCLC	0	0	0	60	60	60	0	20
88	42	male	non-smoker	chemotherapy plus radiotherapy	3	SCLC	SCLC	0	0	0	10	10	10	1	9
89	70	female	non-smoker	chemotherapy	1	SCLC	SCLC	0	0	0	5	5	5	0	19
90	51	male	non-smoker	chemotherapy	1	SCLC	SCLC	0	0	0	20	20	20	0	19.7
91	60	male	non-smoker	not receive	2	SCLC	SCLC	0	0	0	20	20	20	0	19
92	68	male	smoker	chemotherapy	3	SCLC	SCLC	0	0	0	30	30	30	1	12.1
93	66	male	smoker	chemotherapy plus radiotherapy	1	SCLC	SCLC	80	80	80	30	40	40	0	19
94	72	male	smoker	chemotherapy	1	SCLC	SCLC	0	0	0	20	10	10	0	18.3
95	67	male	smoker	chemotherapy plus radiotherapy	1	SCLC	SCLC	0	0	0	80	80	80	0	18.2
96	63	female	non-smoker	chemotherapy plus radiotherapy	1	SCLC	SCLC	0	0	0	20	20	20	0	18
97	63	male	non-smoker	chemotherapy	2	SCLC	SCLC	0	0	0	5	5	5	1	11.3
98	78	male	smoker	chemotherapy	1	SCLC	SCLC	0	0	0	30	30	30	0	17
99	51	male	smoker	chemotherapy plus radiotherapy	1	SCLC	SCLC	0	0	0	20	10	10	1	3.2
100	69	male	smoker	not receive	1	SCLC	SCLC	0	0	0	30	30	30	1	6
101	62	male	non-smoker	not receive	1	SCLC	SCLC	0	0	0	5	5	5	0	17
102	61	female	non-smoker	chemotherapy	1	SCLC	SCLC	80	90	80	30	30	30	0	17.9

Abbreviation: Gal-9, galectin-9; RFS, recurrence-free survival; SCLC, small cell lung cancer; TILs, tumor infiltrating lymphocytes.

Table S2. Logistic regression for galectin-9 expression on TILs

	**Univariate**			**Multivariate**		
**Variables**	**OR**	**95%CI**	**p**	**OR**	**95%CI**	**p**
Gender (Female vs. Male)	2.119	0.563-7.970	0.267			
Age (<70 vs. ⩾70)	0.676	0.224-2.039	0.487			
Smoking status (Non-smoker vs. Smoker)	1.467	0.612-3.515	0.390			
Metastasis (negative vs. positive)	0.877	0.087-8.797	0.911			
SCLC staging (I-II vs. III)	0.589	0.235-1.471	0.257			
PD-1 on TILs (negative vs. positive)	4.860	1.922-12.290	**0.001**	1.077	0.282-4.120	0.914
PD-L1 on TILs (negative vs. positive)	8.446	3.177-22.454	**<0.001**	2.820	0.744-10.696	0.127
PD-L1 on tumor cells (negative vs. positive)	5.615	0.489-64.544	0.166			
CD3 (negative vs. positive)	30.727	6.706-140.795	**<0.001**	11.581	2.093-64.083	**0.005**
CD4 (negative vs. positive)	17.229	5.751-51.609	**<0.001**	3.372	0.764-14.890	0.109
CD8 (negative vs. positive)	11.162	3.984-31.268	**<0.001**	1.351	0.318-5.745	0.684
FOXP3 (negative vs. positive)	6.000	1.033-34.844	**0.046**	0.667	0.077-5.766	0.713

Abbreviation: SCLC, small cell lung cancer; TILs, tumor infiltrating lymphocytes; PD-1, program death-1; PD-L1, program death-ligand 1;OR, Odds Ratio; 95% CI, 95% confidence interval; P, P value for whole.

Statistically significant data were marked with bold and underline.

Table S3. Logistic regression for galectin-9 expression on tumor cells

	Univariate			Multivariate		
**Variables**	**OR**	**95%CI**	**p**	**OR**	**95%CI**	**p**
Gender (Female vs. Male)	0.897	0.303-2.650	0.843			
Age (<70 vs. ⩾70)	1.993	0.763-5.205	0.159			
Smoking status (Non-smoker vs. Smoker)	0.713	0.303-1.678	0.438			
Metastasis (negative vs. positive)	2.267	0.305-16.858	0.424			
SCLC staging (I-II vs. III)	0.967	0.413-2.265	0.939			
PD-1 on TILs (negative vs. positive)	0.682	0.281-1.656	0.398			
PD-L1 on TILs (negative vs. positive)	0.681	0.265-1.749	0.425			
PD-L1 on tumor cells (negative vs. positive)	0.000	/	0.999			
CD3 (negative vs. positive)	0.989	0.428-2.287	0.980			
CD4 (negative vs. positive)	1.321	0.510-3.419	0.567			
CD8 (negative vs. positive)	0.752	0.279-2.023	0.572			
FOXP3 (negative vs. positive)	1.100	0.191-6.339	0.915			

Abbreviation: SCLC, small cell lung cancer; TILs, tumor infiltrating lymphocytes; PD-1, program death-1; PD-L1, program death-ligand 1;OR, Odds Ratio; 95% CI, 95% confidence interval; P, P value for whole.

Statistically significant data were marked with bold and underline.

Table S4. Characteristics of stage IV SCLC patients (n=9)

Characteristic	N (%)	Characteristic	N (%)
Gender		T stage	
Female	3(33.3)	2	5(55.6)
Male	6(66.7)	3	2(33.3)
Age, median, years	63	4	2(22.2)
<70	7 (77.8)		
≥70	2(22.2)	N stage	
Postoperative treatment		0	2(22.2)
NA	1(11.1)	1	1(11.1)
Chemotherapy	4 (44.4)	2	4(44.4)
Chemotherapy plus radiotherapy	4(44.4)	3	2(22.2)

Metastasis	
No	0(0.00)
Yes	9(100)

Abbreviation: T, Tumor; N, Lymph Node; NA, not available; SCLC, small cell lung cancer.

Table S5. Relationship between galectin-9 and clinicopathological factors in stage IV SCLC patients

	Galectin-9 expression	
**Factors**	**R**	**P**
PD-1 expression	0.900	**<0.001**
PD-L1 expression	0.931	**<0.001**
CD3	0.830	**<0.001**
CD4	0.860	**<0.001**
CD8	0.687	**0.04**
FOXP3	0.868	**0.002**
Age	-0.316	0.408
Sex	-0.348	0.359

Abbreviation: SCLC, small cell lung cancer; R, Pearson's correlation coefficients; P, p value for whole.

Statistically significant data were marked with bold and underline.
