# Supplementary material for: Galectin-9-based immune risk score model helps to predict relapse in stage I–III small cell lung cancer
Source: J Immunother Cancer. 2020 Oct 20;8(2):e001391. doi: 10.1136/jitc-2020-001391 (PMC7577067; doi:10.1136/jitc-2020-001391)
Supplement: Supplementary data [file jitc-2020-001391supp002.pdf]

Figure legends

Figure 1. The expression of Gal-9 on cancer cells and TILs.

Abbreviation: Gal-9, galectin-9; TILs, tumor infiltrating lymphocytes.

Figure 2. Survival analysis by Gal-9 level on tumor cells and TILs.

Abbreviation: Gal-9, galectin-9; TILs, tumor infiltrating lymphocytes; P, P value for whole; RFS, recurrence-free survival.

Figure 3. Survival analysis by Gal-9 level on TILs in combination with PD-1 or PD-L1.

Abbreviation: Gal-9, galectin-9; PD-1, program death-1; PD-L1, program death-ligand 1; P, P value for whole; RFS, recurrence-free survival; TILs, tumor infiltrating lymphocytes; TCs, tumor cells.

Figure 4. Prognostic performance of risk score model in SCLC. A) Importance analysis of all immune biomarkers in SCLC by XGBoost algorithm. The diagram of feature importance to outcome illustrated that Gal-9 on TLLs ranked first, CD4 second, and PD-L1 on TILs third. B) Log-rank test results showed that the predictive curve was fitted well with the actual one in SCLC (21.000 months, 95% CI, 16.326-25.674, Vs. 17.000 months, 95% CI, 9.692-24.308, p=0.300). C) Survival analysis of risk score. D) Time-dependent ROC curves and AUC values for the risk

score model and single immune biomarker. The AUC value for risk score model, Gal-9 on TLLs, CD4, and PD-L1 on TILs were 0.671, 0.622, 0.621, 0.644, respectively. Abbreviation: Gal-9, galectin-9; PD-1, program death-1; PD-L1, program death-ligand 1; SCLC, small cell lung cancer; TILs, tumor infiltrating lymphocytes; XGBoost, eXtreme Gradient Boosting algorithm.

Figure 5. Gene set enrichment analysis of Gal-9 expression in SCLC. The top four significant enrichment plots in high Gal-9 expression group compared with that in low Gal-9 expression group. Abbreviation: Gal-9, galectin-9; SCLC, small cell lung cancer.

Figure 6. The landscapes of immune infiltration in SCLC patients with high and low risk. A) Relative percentage of immune infiltration in high-risk SCLC patients. B) Relative percentage of immune infiltration in SCLC patients with low-risk. Abbreviation: SCLC, small cell lung cancer.

Figure S1. Survival analysis by Gal-9 level on TILs in combination with other immune checkpoints.

Abbreviation: Gal-9, galectin-9; P, P value for whole; RFS, recurrence-free survival; TILs, tumor infiltrating lymphocytes; TCs, tumor cells.

Figure S2. Survival analysis by Gal-9 level on tumor cells in combination with other immune checkpoints.

Abbreviation: Gal-9, galectin-9; P, P value for whole; RFS, recurrence-free survival; TILs, tumor infiltrating lymphocytes; TCs, tumor cells.

Figure S3. Clinical value of Gal-9 and risk score model in advanced SCLC. A) Relation between Lgals9 and other factors, including immune checkpoints and clinical features.

B) Survival analysis by Gal-9 level in advanced SCLC. C) Survival analysis by Gal-9-based immune risk score in advanced SCLC.

Abbreviation: Gal-9, galectin-9; P, P value for whole; PD-1, program death-1; PD-L1, program death-ligand 1; SCLC, small cell lung cancer.

Figure S4. Validation of Gal-9 expression level in SCLC. A) According to the data from CCLE, Lgals9 was lowly expressed in the SCLC cell lines compared with NSCLC cell lines. B) Violin plots visualizing significant differences of Lgals9 expression between SCLC and normal tissues in both GSE43346 (p=0.014) and GSE6044 (p=0.028) dataset. Abbreviation: CCLE, Cancer Cell Line Encyclopedia; Gal-9, galectin-9; GEO, Gene Expression Omnibus; NSCLC, non small cell lung cancer; P,

P value for whole; SCLC, small cell lung cancer.

Figure S5. Analysis of top four Gal-9-related pathways in SCLC. A) A total of 15 genes overlapped in three of these pathways. B) Four gene sets with ES > 0.60 and FDR< 0.25 were chosen for the leading edge analysis. The Jaccard values of numbers of the occurrences mainly concentrated on the range of 0-0.40. Abbreviation: DEGs, differentially expressed genes; ES, enrichment scores; FDR, false discovery rates; Gal-9, galectin-9; SCLC, small cell lung cancer.

Figure S6. Gene set enrichment analysis of Gal-9 expression in SCLC. A) Violin plots visualizing 18 Gal-9-associated genes were differentially expressed between normal and SCLC tissues. B) The correlation matrix of all 18 DEGs and Lgals9 that encoding Gal-9. C) Visualization of the strong correlation (>0.6) between Gal-9 and DEGs by Cytoscape. Abbreviation: DEGs, differentially expressed genes; Gal-9, galectin-9; P, P value for whole; SCLC, small cell lung cancer.

Figure S7. Relationship between immune cell proportions in SCLC patients with high and low risk. A) Correlation matrix of all immune cell proportions in SCLC patients with high-risk. B) Correlation matrix of all immune cell proportions in low-risk SCLC patients. C) Violin plots

visualizing significantly different activated memory CD4 T cells between high-risk and low-risk SCLC patients. Abbreviation: P, P value for whole; SCLC, small cell lung cancer.
