# Supplementary figures and images for: Galectin-9-based immune risk score model helps to predict relapse in stage I–III small cell lung cancer
Source: J Immunother Cancer. 2020 Oct 20;8(2):e001391. doi: 10.1136/jitc-2020-001391 (PMC7577067; doi:10.1136/jitc-2020-001391)

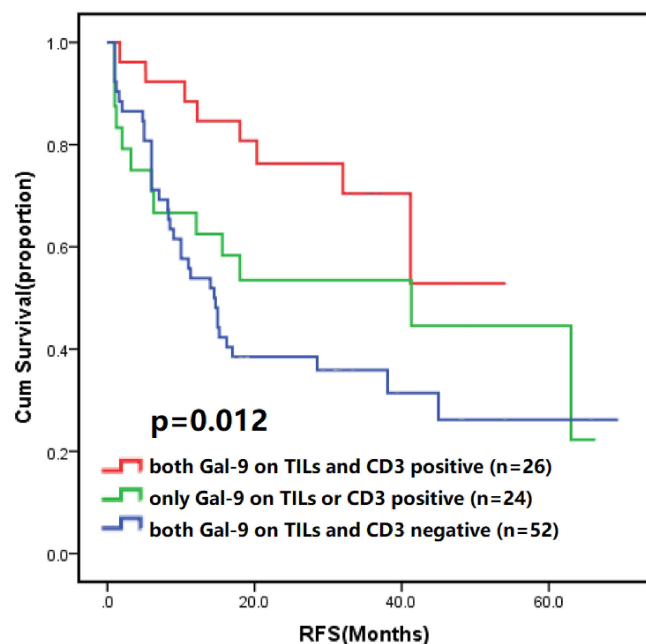

A) Gal-9 on TILs and CD3

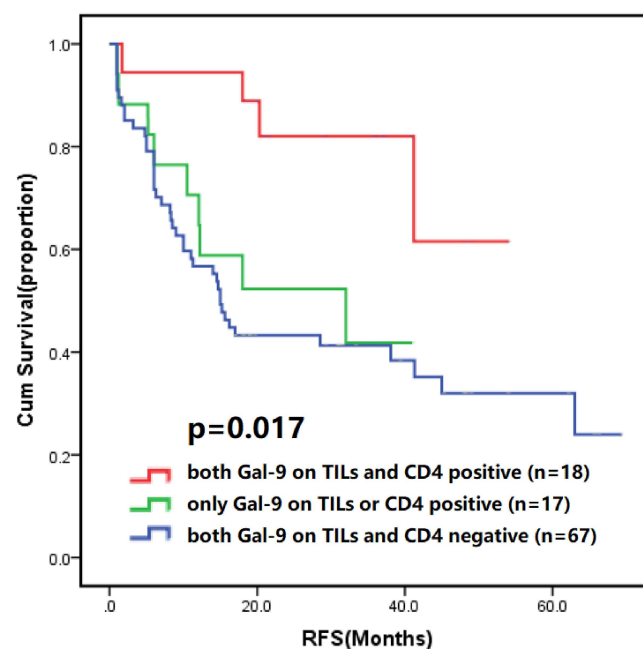

B) Gal-9 on TILs and CD4

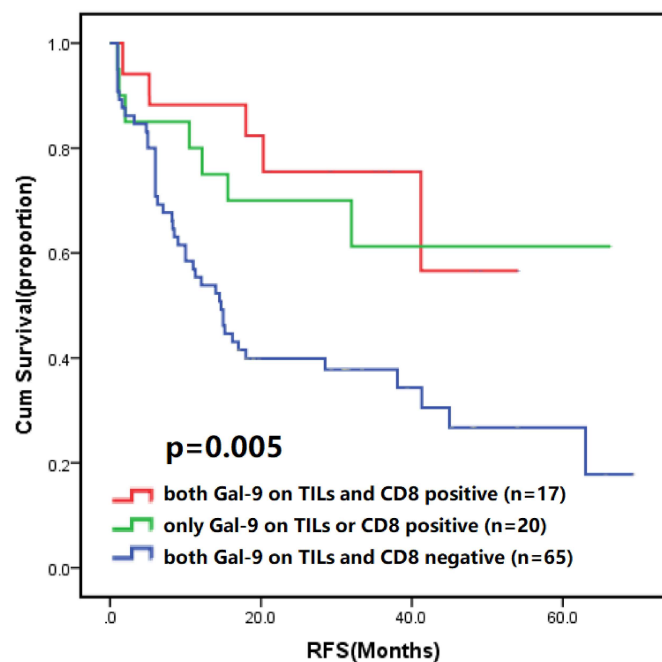

C) Gal-9 on TILs and CD8

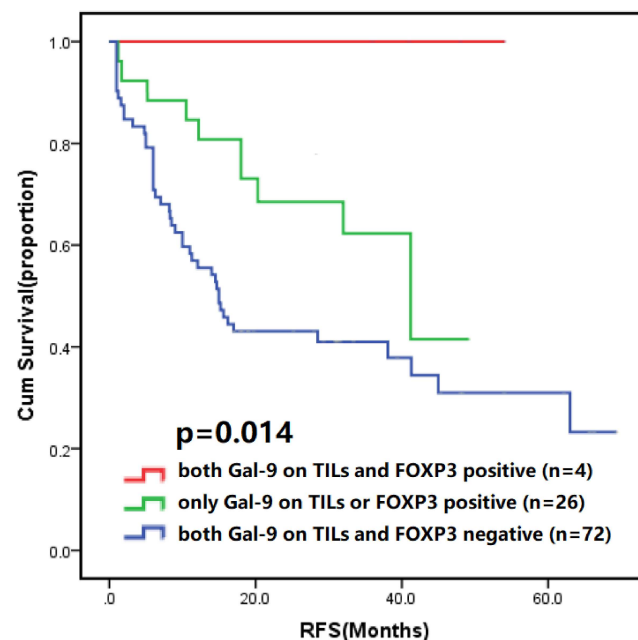

D) Gal-9 on TILs and FOXP3

Supplement: Supplementary data [file jitc-2020-001391supp003.pdf]

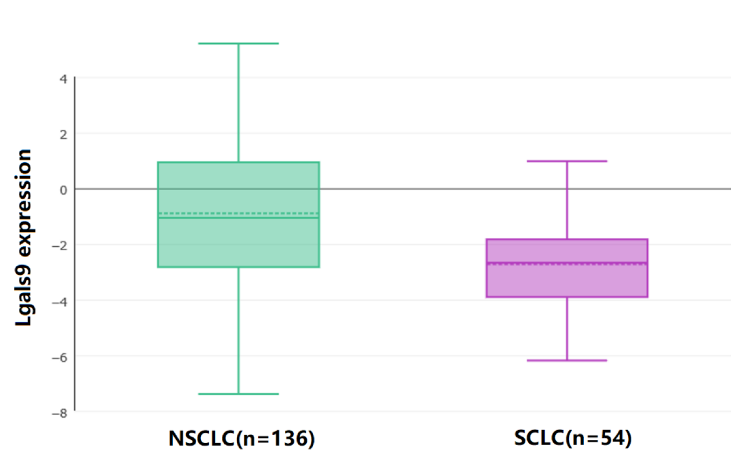

A) CCLE database

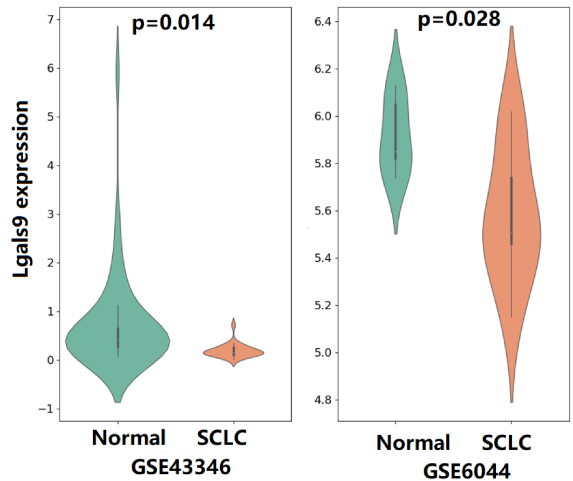

B) GEO database

Supplement: Supplementary data [file jitc-2020-001391supp006.pdf]

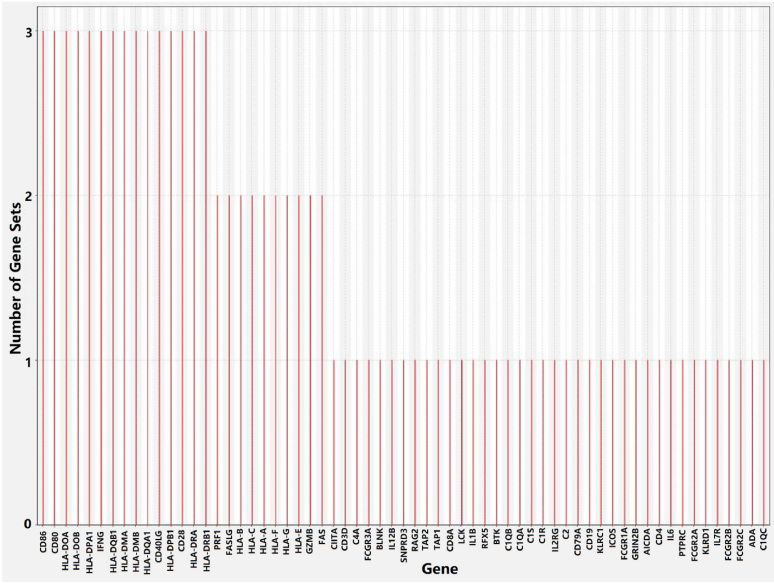

A) Overlapping genes in Gal-9-related pathways.

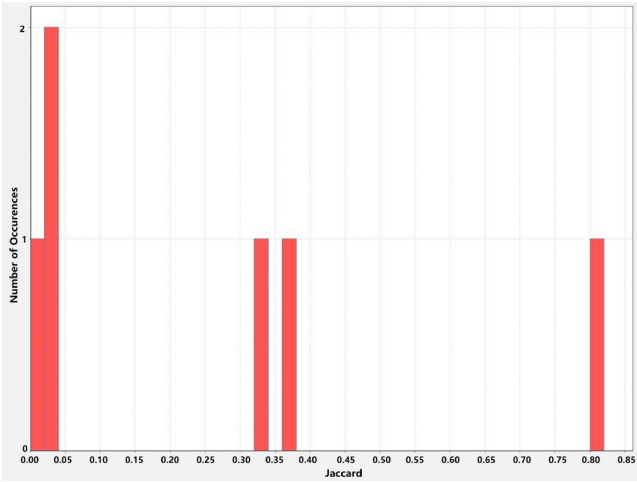

B) The leading edge analysis.

Supplement: Supplementary data [file jitc-2020-001391supp007.pdf]
