# Supplementary material for: Galectin-9-based immune risk score model helps to predict relapse in stage I–III small cell lung cancer
Source: J Immunother Cancer. 2020 Oct 20;8(2):e001391. doi: 10.1136/jitc-2020-001391 (PMC7577067; doi:10.1136/jitc-2020-001391)
Supplement: Supplementary data [file jitc-2020-001391supp004.pdf]

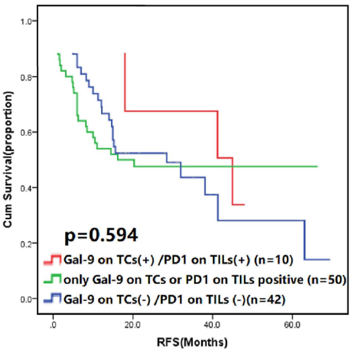

A) Gal-9 on TCs and PD1 on TILs

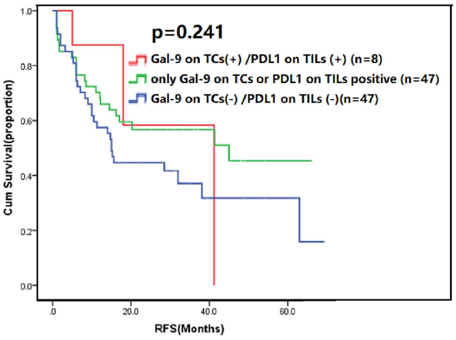

B) Gal-9 on TCs and PDL1 on TILs

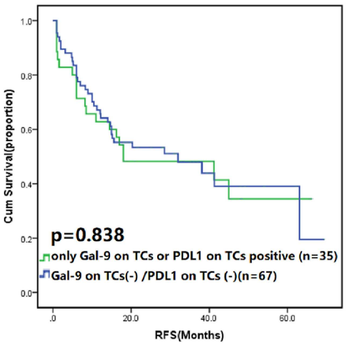

C) Gal-9 on TCs and PDL1 on tumor cells

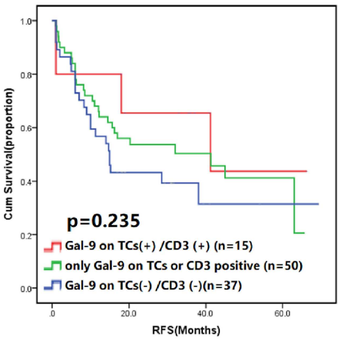

D) Gal-9 on TCs and CD3

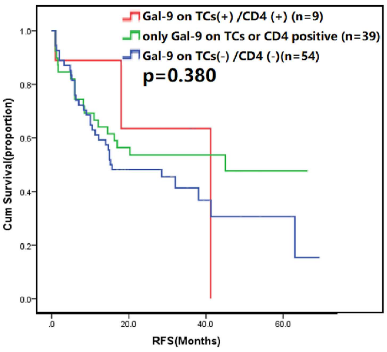

E) Gal-9 on TCs and CD4

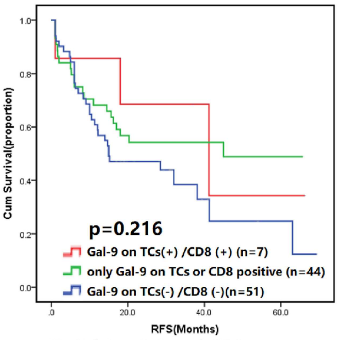

F) Gal-9 on TCs and CD8

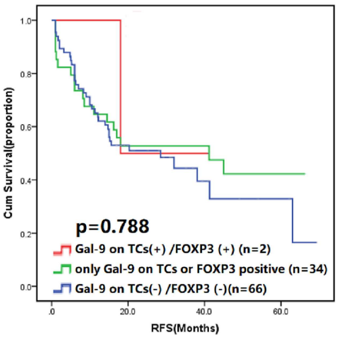

G) Gal-9 on TCs and FOXP3
