# Supplementary material for: Galectin-9-based immune risk score model helps to predict relapse in stage I–III small cell lung cancer
Source: J Immunother Cancer. 2020 Oct 20;8(2):e001391. doi: 10.1136/jitc-2020-001391 (PMC7577067; doi:10.1136/jitc-2020-001391)
Supplement: Supplementary data [file jitc-2020-001391supp008.pdf]

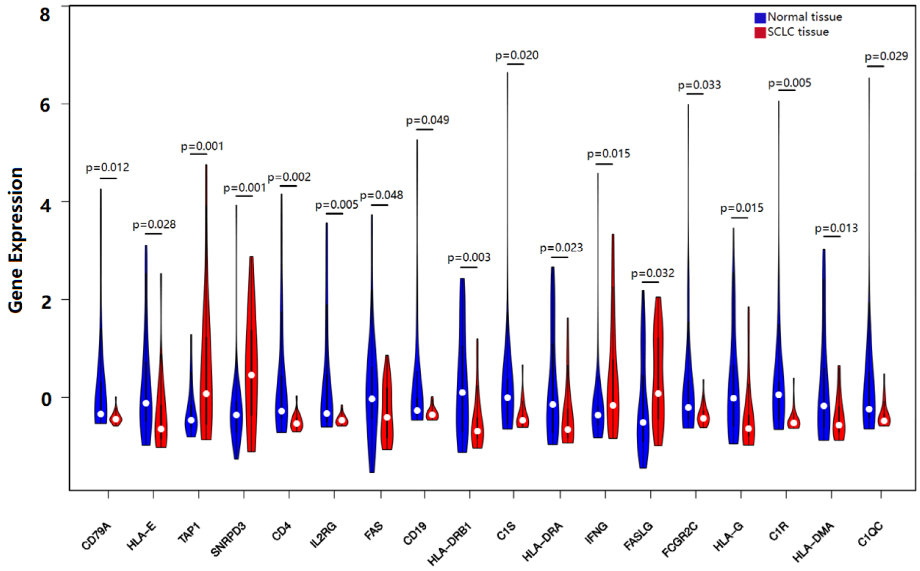

A) Validation of Gal-9-associated genes expression in SCLC

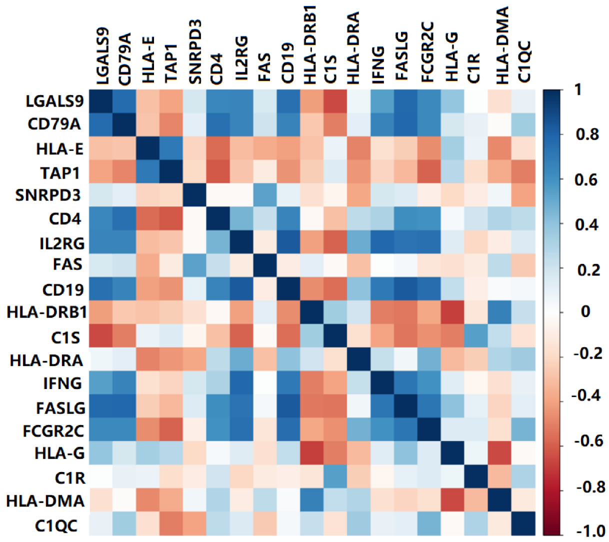

B) Relation between Lgals9 and 18 differentially expressed genes

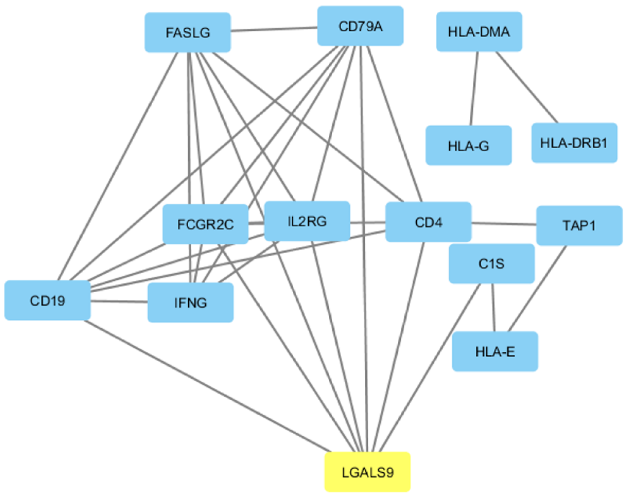

C) The Lgals9-associated network by Cytoscape
