# Supplementary material for: Galectin-9-based immune risk score model helps to predict relapse in stage I–III small cell lung cancer
Source: J Immunother Cancer. 2020 Oct 20;8(2):e001391. doi: 10.1136/jitc-2020-001391 (PMC7577067; doi:10.1136/jitc-2020-001391)
Supplement: Supplementary data [file jitc-2020-001391supp009.pdf]

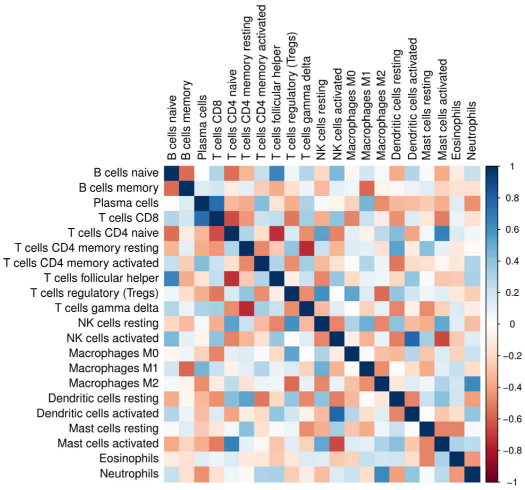

A) Relationship between immune cell proportions in high risk

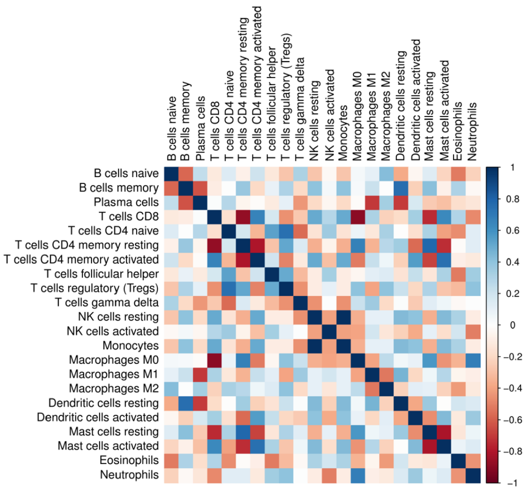

B) Relationship between immune cell proportions in low risk

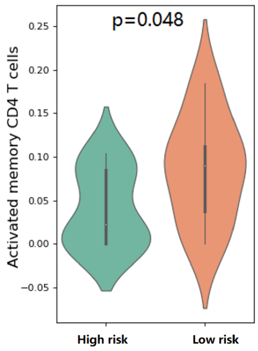

C) Activated memory CD4 T cells
